# Roles for HB‐EGF in Mesenchymal Stromal Cell Proliferation and Differentiation During Skeletal Growth

**DOI:** 10.1002/jbmr.3596

**Published:** 2018-12-14

**Authors:** Ping Li, Qi Deng, Jiajia Liu, Jianshe Yan, Zhanying Wei, Zhenlin Zhang, Huijuan Liu, Baojie Li

**Affiliations:** ^1^ Bio‐X Institutes Key Laboratory for the Genetics of Developmental and Neuropsychiatric Disorders Ministry of Education Shanghai Jiao Tong University Shanghai China; ^2^ School of Life Sciences Shanghai University Shanghai China; ^3^ Shanghai Institute of Immunology Department of Immunology and Microbiology Shanghai Jiao Tong University School of Medicine Shanghai China; ^4^ Metabolic Bone Disease and Genetic Research Unit Department of Osteoporosis and Bone Diseases Shanghai Key Clinical Center for Metabolic Disease Shanghai Jiao Tong University Affiliated Sixth People's Hospital Shanghai China

## Abstract

HB‐EGF, a member of the EGF superfamily, plays important roles in development and tissue regeneration. However, its functions in skeletal stem cells and skeleton development and growth remain poorly understood. Here, we used the Cre/LoxP system to ablate or express HB‐EGF in Dermo1+ mesenchymal stromal cells and their progenies, including chondrocytes and osteoblast lineage cells, and bone marrow stromal cells (BMSCs). *Dermo1‐Cre; HB‐EGF^f/f^* mice only showed a modest increase in bone mass, whereas *Dermo1‐HB‐EGF* mice developed progressive chondrodysplasia, chondroma, osteoarthritis‐like joint defects, and loss of bone mass and density, which were alleviated by treatment with EGFR inhibitor AG1478. The cartilage defects were recapitulated in chondrocyte‐specific HB‐EGF overexpression (*Col2‐HB‐EGF*) mice with a lesser severity. *Dermo1‐HB‐EGF* mice showed an increase in proliferation but defects in differentiation of chondrocytes and osteoblasts. HB‐EGF promoted BMSC proliferation via the Akt1 and Erk pathways but inhibited BMSC differentiation via restraining Smad1/5/8 activation. However, *Dermo1‐HB‐EGF* mice showed normal osteoclastogenesis and bone resorption. These results reveal an important function of autocrine or paracrine HB‐EGF in mesenchymal stromal cell proliferation and differentiation and suggest that EGF signaling needs to be tightly controlled to maintain bone and articular cartilage integrity. © 2018 The Authors. *Journal of Bone and Mineral Research* Published by Wiley Periodicals Inc.

## Introduction

Heparin‐binding EGF‐like growth factor (HB‐EGF) is a member of the EGF superfamily that also includes EGF, TGFα, and amphiregulin, with HB‐EGF having high affinity for heparin. HB‐EGF is cloned from monocytes and macrophages and is synthesized as a membrane‐anchored glycoprotein.^1^ Ectodomain shedding by proteases generates the soluble form of HB‐EGF, which can function in autocrine and paracrine fashions.^2^, ^3^ The membrane‐bound HB‐EGF can function in juxtacrine signaling. Both soluble and membrane‐bound HB‐EGF participates in normal physiological processes.^4^, ^5^ HB‐EGF binds to EGF receptor (EGFR, ErbB1) and ErbB4, leading to activation of Akt1, Erk, and other pathways to promote cell proliferation, survival, and migration.^6^, ^7^ Interestingly, membrane‐bound HB‐EGF also serves as a receptor for diphtheria toxin (DTR).^8^

Gene knockout mouse studies have shown that HB‐EGF is essential for mouse survival. *HB‐EGF^‐/‐^* mice show neonatal lethality with dilated cardiac chambers and eyelid defects.^9^, ^10^, ^11^ In addition, HB‐EGF takes part in skin wound healing,^12^ liver regeneration,^13^ and gut mucosa restitution.^14^, ^15^ HB‐EGF is also involved in pathological processes such as tumor progression and metastasis;^16^ for example, transgenically expressed HB‐EGF is tumorigenic in pancreas.^17^ All these studies point to pro‐proliferation and anabolic roles for HB‐EGF.

HB‐EGF signaling has been implicated in skeletal development, which involves coordinated bone formation and resorption.^18^ Although cell line‐based studies suggest that HB‐EGF promotes osteoblast proliferation but inhibits differentiation,^19^, ^20^ in vivo functions of HB‐EGF remain underexplored. On the other hand, the roles of EGFR, one of two receptors of HB‐EGF, in skeletal development have been examined in several studies. Knockout of EGFR results in delayed formation of the secondary ossification center and recruitment of osteoblasts and osteoclasts, leading to osteoporosis.^21^ Although osteoblast‐specific EGFR knockout mice (*Col1‐Cre; EGFR^f/f^*) showed no skeletal phenotypes, overexpression of a dominant negative EGFR mutant in osteoblasts led to a decrease in the number of osteoblasts and an increase in the number of osteoclasts, resulting in osteoporosis.^22^ In addition, overexpression of the dominant negative EGFR in Col2+ chondrocytes leads to defects in chondrocyte proliferation and survival and development of osteoarthritis, and EGFR signaling has been proposed as a target to prevent osteoarthritis development.^23^, ^24^, ^25^ These results suggest that EGFR signaling plays an anabolic role in skeletal development through promoting proliferation of osteoblasts and chondrocytes. However, a recent study showed that HB‐EGF was upregulated in osteoarthritis cartilage samples and HB‐EGF promoted the catabolic activities of articular chondrocytes.^26^ Thus, the roles of HB‐EGF signaling in development of the skeleton and the etiology of related diseases remain unclear and need further investigation.

Here, we found that HB‐EGF was highly expressed in bone marrow stromal cell (BMSC) cultures compared with BMSC‐derived osteoblasts and chondrocytes. We used the Cre/LoxP system to ablate or overexpress HB‐EGF in Dermo1 lineage cells to examine its roles in skeletal development and growth in comparison with chondrocyte‐specific HB‐EGF overexpression mouse models. Although *HB‐EGF* knockout only modestly increased the bone mass, increased expression of HB‐EGF in Dermo1 lineage cells suppressed osteogenic and chondrogenic commitment/differentiation, which led to bone and cartilage defects, respectively. Our findings reveal critical roles of HB‐EGF in regulating proliferation and differentiation of osteoblasts, chondrocytes, and BMSC cultures in autocrine and/or paracrine manners and elucidate the mechanisms by which HB‐EGF executes its functions in skeletal growth and in pathogenesis of osteoarthritis.

## Materials and Methods

Mouse work was carried out following the recommendations from the National Research Council Guide for the Care and Use of Laboratory Animals, with the protocols approved by the Institutional Animal Care and Use Committee of Shanghai, China (SYXK [SH] 2011‐0112). *Dermo1‐Cre*, *Col2‐Cre*, and *ROSA‐tdTomato* mouse lines were purchased from the Jackson Laboratory (Bar Harbor, ME, USA). *Rosa‐STOP‐DTR* mice were generated in Waisman's laboratory and floxed *HB‐EGF* mouse was generated from Mekada's laboratory. These mice were kept at the SPF facility of Shanghai Jiao Tong University. *HB‐EGF^f/f^* mice were crossed with *Dermo1‐Cre* mice to generate *Dermo1‐Cre; HB‐EGF^f/f^* mice. *Dermo1‐Cre* and *Col2‐Cre* mice were crossed with *Rosa‐STOP‐DTR* mice to generate *Dermo1‐HB‐EGF* mice and *Col2‐HB‐EGF* mice, respectively. Dermo1‐Cre mice were crossed with *ROSA‐tdTomato* mice to generate *Dermo1‐tdTomato* mice.

### Isolation and culture of BMSCs

For BMSC isolation, 8‐week‐old mice were euthanized and the femur and tibia were extracted and cleaned. The bone ends were cut off and the bone marrow was flushed out with α‐MEM. Single‐cell suspension was generated through pepiting and was filtered through a 70 μm mesh to remove debris. BMSCs were cultured in α‐MEM containing 15% FBS, 100 μg/mL penicillin, and 100 μg/mL streptomycin, at 37°C for 5 days. The nonadherent cells were washed out and the BMSCs were used for proliferation and differentiation experiments. To get Dermo1 lineage BMSCs, femur and tibia bones were extracted from *Dermo1‐Cre; tdTomato* mice and Tomato+ BMSCs were sorted out with FACS.

### Differentiation of BMSCs

To induce BMSC tri‐lineage differentiation, sorted Dermo1 lineage BMSCs were seeded at 5 × 10^4^/well in 12‐well plates. The next day, the cells were switched into osteogenic medium with α‐MEM medium containing 15% FBS, 10 mM β‐glycerol phosphate, and 50 μg/mL ascorbic acid, for 7 to 10 days, with medium changed every 2 days. The cells were then fixed in 4% paraformaldehyde and stained for ALP using an Alkaline Phosphatase Kit (Sigma‐Aldrich, St. Louis, MO, USA) or Alizarin red. For mineralization assay, the cells were cultured for 21 or 28 days, which were stained in 5% silver nitrate solution under ultraviolet light or 1% Alizarin red S. The silver staining was terminated by adding sodium thiosulfate solution. For adipocyte differentiation, BMSCs were plated at 1 × 10^5^/well in a 12‐well plate and cultured in α‐MEM containing 15% FBS, 100 nM dexamethasone, and 5 μM insulin for 2 weeks. The cells were then fixed and stained with Oil‐red‐O solution. For chondrocyte differentiation, BMSCs were seeded at 1 × 10^5^/well in 12‐well plates, cultured in α‐MEM containing 15% FBS, 100 nM dexamethasone, 10 ng/mL TGFβ1, and 1 μM ascorbate‐2‐phosphate for 3 weeks, which were then fixed and stained with 1% Alcian blue or Toluidine blue or harvested for RNA isolation.

### Pellet culture assays (chondrogenesis)

We first sorted out Dermo1‐Tomato BMSCs with FACS and suspended them in chondrogenic medium consisting of high‐glucose DMEM supplemented with 10 ng/mL recombinant human transforming growth factor‐β3 (TGF‐β3; R&D Systems, Minneapolis, MN, USA), 100 nM dexamethasone (Sigma), 50 µg ascorbic acid 2‐phosphate/mL (Sigma), 1 mM sodium pyruvate, 40 µg proline/mL, and ITS + premix (Sigma; final concentrations: 6.25 µg/mL bovine insulin, 6.25 µg/mL transferrin, 6.25 µg/mL selenous acid, 5.33 µg/mL linoleic acid, and 1.25 mg/mL bovine serum albumin). Aliquots of 2.5 × 10^5^ cells, suspended in 500 µL chondrogenic medium, were centrifuged at 300*g* for 5 minutes in 15 mL polypropylene conical tubes. Pelleted cells were incubated at 37°C under 5% CO_2_ with loosened caps to permit gas exchange. Within 24 hours of incubation, the sedimented cells formed a spherical aggregate at the bottom of the tube. The medium was changed every 3 days and pellets were harvested on 6 weeks.

### Micromass culture assays (chondrogenesis)

For micromass culture‐based chondrogenesis assays, sorted Dermo1 lineage BMSCs were suspended at a concentration of 1.6 × 10^7^ cells/mL. We generated micromass cultures by seeding 10 μL droplets of cell suspension at the center of 12‐well plates. Cells were allowed to attach for 2 hours before adding α‐MEM containing 15% FBS, 100 IU/mL penicillin, and 100 µg/mL streptomycin. After 1 day, the medium was replaced with chondrogenic medium in α‐MEM containing 15% FBS, 100 nM dexamethasone, 10 ng/mL TGFβ1, and 1 μM ascorbate‐2‐phosphate. Cultures were maintained for 21 days, with the medium changed every 3 days, and were lastly stained with Toluidine blue.

### Isolation and differentiation of monocyte

The femur and tibia bones were extracted from adult mice and the bone marrow was flushed out with α‐MEM. The cells were dispersed and filtered through a 70 μm mesh, which were pelleted. The red blood cells were lyzed and the bone marrow cells were washed and cultured in α‐MEM containing 15% FBS, 100 μg/mL penicillin, 100 μg/mL streptomycin at 37°C for 16 hours. The nonadherent cells were harvested and seeded at 1 × 10^6^ cells/well in 96‐well plates for TRAP staining. These cells were cultured in α‐MEM medium containing M‐CSF (50 ng/mL) and RANKL (100 ng/mL) for 5 days, and then stained for TRAP using a TRAP‐leukocyte kit (Sigma‐Aldrich), following the manufacturer's instructions.

### TRAP staining of bone sections

To stain bone sections for TRAP‐positive osteoclasts, all bone sections were deparaffinized in xylene and rehydrated in decreasing concentrations of alcohol. The bone sections were then stained with the Leukocyte Acid Phosphatase Kit (Sigma‐Aldrich) for 1 hour at 37°C following the manufacturer's instructions. Images were taken under Olympus DP72 microscope (Olympus Microsystems, Tokyo, Japan).

### Quantitative PCR

Total RNA was extracted using Trizol regent (Invitrogen, Carlsbad, CA, USA), which was reverse transcribed using Transcriptor Universal cDNA Master (Roche, Mannheim, Germany) following the manufacturer's instructions. Quantitative PCR was carried out using Fast Start Universal SYBR Green Master kit (Roche) on ABI Prism 7500 Sequence Detection System (Applied Biosystems, Carlsbad, CA, USA). The levels of different mRNA species were calculated with the delta‐delta CT method and normalized to GAPDH. Significant difference was analyzed using two‐tailed Student's *t* test. The PCR consists of 10 seconds for denaturation at 95°C, 15 seconds for annealing at 67°C, and 10 seconds for extension at 72°C. The primer sequences and GenBank accession numbers are listed in Supplemental Table S1.

### Bone decalcification

Femur and tibiae bones were fixed in 4% PBS‐buffered paraformaldehyde overnight at 4°C, and decalcified in 15% EDTA for 2 weeks. The samples were dehydrated in alcohol, cleared with xylene, and embedded in paraffin. Four‐µm‐thick sections were cut using microtome (Leica Microsystems, Wetzlar, Germany). Hematoxylin and eosin (H/E) or safranin O staining was carried out on bone sections as previously described.^27^ Stained slides were photographed under a light microscope (Olympus Microsystems).

### Histomorphometry

Bone histomorphometry was performed on undecalcified sections. For dynamic histomorphometric analysis, mice received injections of calcein (20 mg/kg) at 8 and 2 days before euthanization. The femur was embedded in methyl methacrylate after gradient alcohols and xylene treatment. Four‐µm‐thick sections were cut using Leica Microsystems microtome. The sections were left unstained for measurement of double calcein labeling. For bone parameter measurement, the slides were stained with Villanueva‐Goldner's trichrome method. Images were taken and analyzed using the Olympus DP72 microscope (Olympus Microsystems). All bone‐specific parameters were measured and expressed in units following the guidelines established by the American Society for Bone and Mineral Research histomorphometry nomenclature committee using OsteoMeasure software (OsteoMetrics, Decatur, GA, USA).

### Immunofluorescence and immunohistochemical staining

Bone sections were deparaffinized in xylene, rehydrated in ethanol, and permeabilized with 0.1% Triton X‐100 for 20 minutes at room temperature. Antigen retrieval was performed in a citrate buffer. For immunostaining, the bone slides or cells on cover glass were blocked with 10% goat serum for 60 minutes, incubated with primary antibodies overnight at 4°C, washed in PBS, incubated with secondary antibody for 1 hour at 37°C, and washed with PBS before mounted on ProLong Gold DAPI (Life Technologies, Carlsbad, CA, USA). The antibodies used in this study were: Col1α (ab21286, Abcam [Cambridge, MA, USA], 1:100), Col2 (ab34712, Abcam, 1:200), Col10 (ab58632, Abcam, 1:100), Non‐immune immunoglobulin G (IgG) (of the same species as the primary antibodies) was used as negative controls. The secondary antibodies were goat anti‐rabbit Alexa Fluor 488 (ThermoFisher Scientific [Waltham, MA, USA], 1:200). Slides were mounted with antifade mounting medium with DAPI (ThermoFisher Scientific). Images were taken under Olympus DP72 microscope (Olympus Microsystems).

For immunohistochemical staining, endogenous peroxidase activity was quenched with 3% H_2_O_2_ in methanol for 20 minutes followed by washing with PBS before primary antibody incubation. After incubation with secondary antibody, sections were developed with diaminobenzidine and counterstained with hematoxylin and then dehydrated and mounted in neutral resins. Primary antibodies were: Ki67 (ab15580, Abcam, 1:100), p‐Akt (CST, 4060S, 1:100), p‐Erk (CST, 4376s, 1:200), FSP1 (ab27957, Abcam, 1:200). Non‐immune immunoglobulin G (IgG) (of the same species as the primary antibodies) was used as negative controls.

### Western blot

Total proteins were extracted from cells or tissues with TNEN buffer containing phosphatase and proteinase inhibitors, quantitated by the Bradford method (Bio‐Rad [Hercules, CA, USA] assay), and subjected to SDS‐PAGE gel electrophoresis, which were transferred onto nitrocellulose membranes. The proteins were detected with specific antibodies using standard Western blot method. The following antibodies were used: β‐Actin (Santa Cruz [Dallas, TX, USA], sc‐47778), HB‐EGF (Santa Cruz, sc‐365182), p‐Akt1 (CST, 9271S), Akt1 (CST, 9272S), p‐Erk (CST, 4377), Erk (CST, 9102S), p‐EGFR (CST, 3777s), EGFR (Abcam, ab5644), p‐Smad1/5/8 (CST, 9511l), Smad1 (CST, 9473l). Immunoreactivity was detected using a Western Chemiluminescent HRP Substrate Kit (Millipore, Burlington, MA, USA) and imaged with FluorChem M system (ProteinSimple, San Jose, CA, USA).

### X‐ray and micro‐CT analysis

The whole‐body and femur radiographs were taken using Cabinet X‐Ray system (LX‐60, Faxitron Bioptics, Tucson, AZ, USA) with standardized settings (45 Kv for 8 seconds), as previously described.^28^ Quantitative analysis was performed in mice femora on a SkyScan‐1176 micro‐CT Scanner (Bruker microCT, Kontich, Belgium), following the procedures provided by the manufacturer. Briefly, scanning was performed using 8.96 μm voxel size, 45 KV, 500 μA, and 0.6 degrees rotation step (180 degrees angular range) through the whole length of the femora and extended proximally for 1400 slices. We started morphometric analysis with the first slice, which the femoral condyles were fully merged and extended for 150 slices proximally. Using a contouring tool, we segmented the trabecular bone from the cortical shell manually on key slices and morphed the contours automatically to segment the trabecular bone on all slices. The three‐dimensional structure and morphometry were constructed and analyzed for bone volume per tissue volume (BV/TV; %), bone mineral density (BMD; mg HA/mm^3^), trabecular number (Tb.N.; mm^–1^), trabecular thickness (Tb.Th.; mm), and trabecular spacing (Tb.Sp; mm). We also performed micro‐CT imaging in the mid‐diaphysis of the femur and performed midshaft evaluation of 100 slices to quantify the cortical thickness, bone mineral density, and outer/inner perimeter of the mid‐shaft.

### In vivo ectopic bone formation assay

A total of 2 × 10^6^ isolated BMSCs were collected and incubated with 40 mg hydroxyapatite/tricalcium phosphate carrier (HA/TCP: 12.5:87.5 by weight) (Bioengineering Research Center of Sichuan University, China) scaffolds for 6 hours at 37°C in humidifying incubator and then implanted subcutaneously onto the back of 2‐month‐old BALB/C homozygous nude (nu/nu) mice (4 mice per group). Mice were euthanized 6 weeks later after transplantation. The implants were fixed in 4% paraformaldehyde and then decalcified for 10 days. The sections (4 μm thick) were stained with H/E. To quantify the bonelike tissues, 10 images of each sample were taken randomly to measure the area of new bone formation versus total area.

### Statistical analyses

Numerical data and histograms were expressed as the mean ± SD. Comparisons between 2 groups were analyzed using 2‐tailed unpaired Student's *t* test. A *p* value < 0.05 was considered statistically different (*p < 0.05 and **p < 0.01). Analysis of mice was litter‐based, and at least three litters were analyzed for every parameter. All the experiments were repeated at least three times.

## Results

### HB‐EGF expression is decreased when BMSCs are induced to differentiate

**Figure 1:**
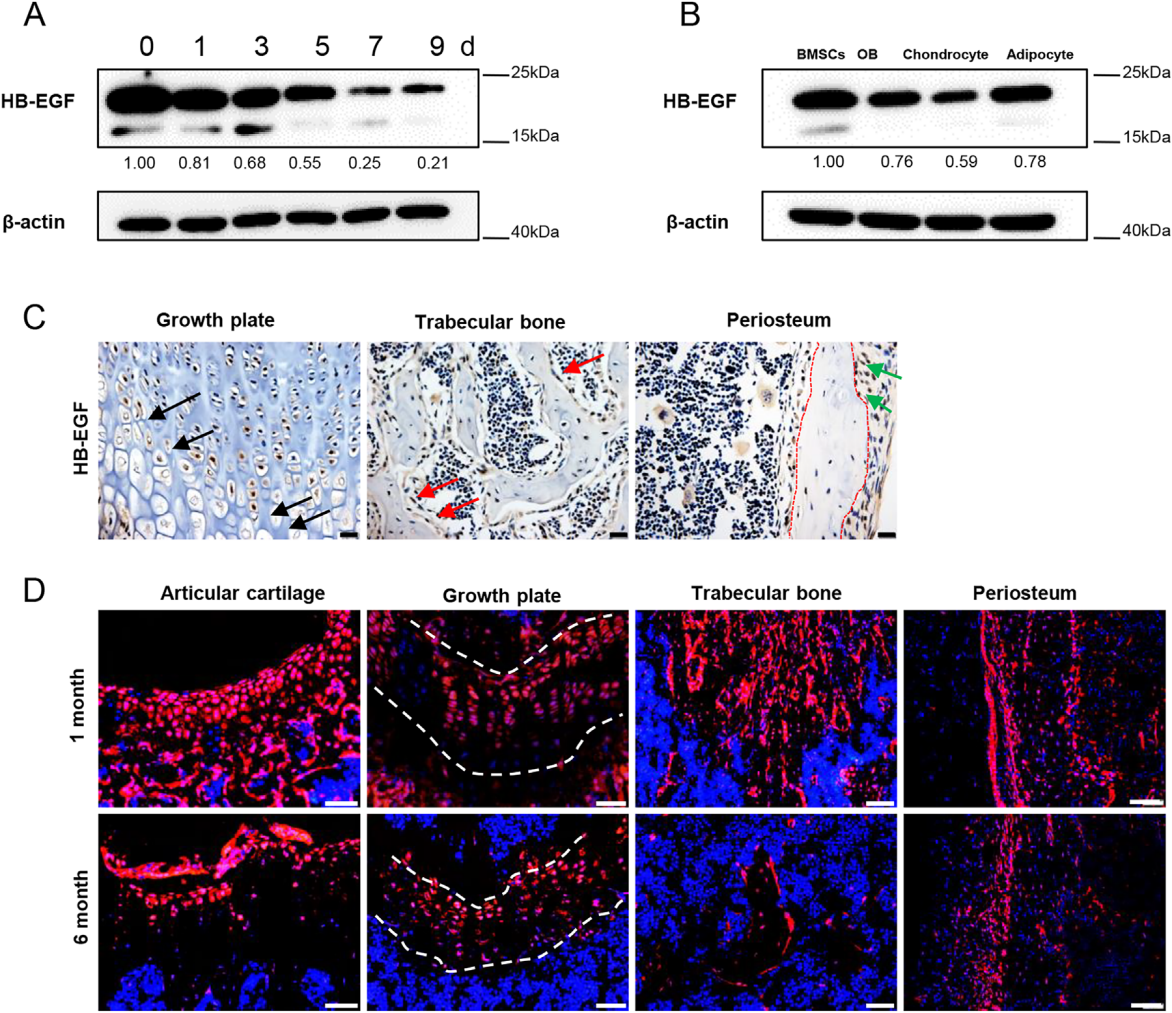
Expression of HB‐EGF is decreased in osteoblasts and chondrocytes compared with BMSCs. (*A*) Western blot results showed that the levels of HB‐EGF were downregulated when BMSCs were induced to differentiate into osteoblasts in vitro. BMSCs were cultured in differentiation medium for different periods of time and the cells were harvested, washed, and lysed for Western blot analysis. The lower bands of HB‐EGF may be truncated forms. The quantitation data of the HB‐EGF bands were shown beneath the gel with the value of day 0 being set at 1.0. (*B*) Western blot results showed that the levels of HB‐EGF were downregulated when BMSCs were induced to differentiate. WT BMSCs were cultured in differentiation medium for osteoblasts, chondrocytes, or adipocytes for 7 days and the cells were collected for Western blot analysis. The lower bands of HB‐EGF may be truncated forms. The quantitation data of the HB‐EGF bands were shown beneath the gel with the value of BMSCs being set at 1.0. (*C*) Immunostaining of HB‐EGF expression on femur section at growth plates, trabecular bones, and periosteum. Scale bar = 20 μm. Arrows indicate chondrocytes or osteoblasts. (*D*) Tracing of Dermo1 lineage cells on bone sections of 1‐ and 6‐month‐old *Dermo1‐Cre; tdTomato* mice. The femur sections were stained with DAPI. Scale bar = 50 µm.

To understand the physiological functions of HB‐EGF in skeletal development and growth, we first determined HB‐EGF expression in BMSC cultures and their differentiated progenies. Western blot results showed that the HB‐EGF levels went down when BMSCs differentiated into osteoblasts in vitro, which was monitored with ALP staining (Fig. 1 *A*, *B* and Supplemental Fig. S1*A*). The levels of HB‐EGF went down as well when BMSCs were induced to differentiate into chondrocytes, which was monitored with Alcian blue staining, but to a lesser extent when induced to differentiate into adipocytes, which was monitored with oil red staining (Fig. 1 *B* and Supplemental Fig. S1*B*). These results suggest that HB‐EGF expression is suppressed when BMSCs start to differentiate. We also immunostained femur bone sections of 1‐month‐old mice with anti‐HB‐EGF antibodies and found that HB‐EGF signals were the strongest at the proliferating zone of the growth plate (Fig. 1 *C* and Supplemental Fig. S1*C*), suggesting that HB‐EGF expression is expressed at high levels in proliferating cells. In addition, HB‐EGF signals were also detected in some osteoblasts at endosteal and periosteal surfaces of cortical bones (Fig. 1 *C*).

Based on the HB‐EGF expression patterns, we attempted to ablate or overexpress HB‐EGF in mesenchymal stromal cells to study its functions in vivo. We chose *Dermo1‐Cre* mice because this knock‐in line has been used to study mesenchymal stromal cells in skeletal development, although it is unclear whether Dermo1 labels stem cells/progenitors for osteoblasts and chondrocytes in adult mice.^29^ We first used *Dermo1‐Cre; ROSA‐tdTomato* mice to trace the Dermo1 lineage at 1 or 6 months of age. Dermo1‐labeled cells were detected in trabecular bone, periosteal surface, growth plate, and articular cartilage (Fig. 1 *D*), suggesting that Dermo1+ cells do give rise to both osteoblasts and chondrocytes. Moreover, we isolated BMSCs from *Dermo1‐Cre; ROSA‐tdTomato* mice and stained them for HB‐EGF and found that these cells also expressed HB‐EGF (Supplemental Fig. S1*D*).

### *Dermo1‐Cre*‐mediated *HB‐EGF* knockout mice show normal skeleton with a slight increase in bone mass

We then generated *Dermo1‐Cre; HB‐EGF^f/f^* mice to ablate *HB‐EGF* in mesenchymal stromal cells.^9^ We found that HB‐EGF expression was decreased by about 80% in BMSCs isolated from *Dermo1‐Cre; HB‐EGF^f/f^* mice (Supplemental Fig. S2*A*). The mutant mice showed normal body weight, body length, femur length, knee joint, growth plate, and articular cartilage at 2 months of age (Supplemental Fig. S2B–F and data not shown). However, the mutant mice showed a modest increase in BMD, bone mass, and the number and thickness of trabeculae (Supplemental Fig. S2G–K). One explanation why *HB‐EGF* ablation leads to modest effects on bone parameters and no effects on cartilage is that the function of HB‐EGF may be made up for by other members of the EGF superfamily.

### Overexpression of HB‐EGF in Dermo1+ cells leads to defects in growth plate and articular cartilage

**Figure 2:**
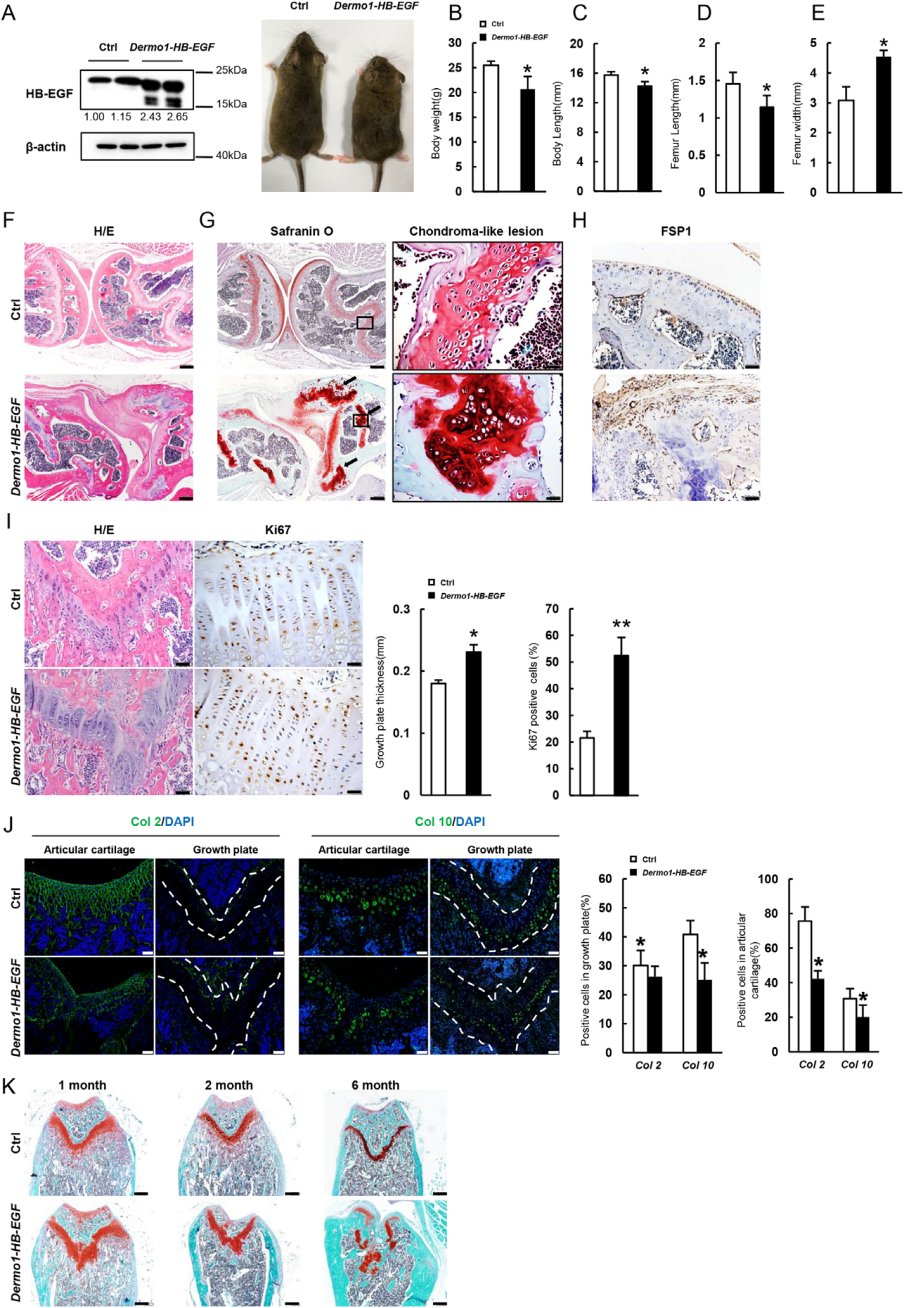
Overexpression of HB‐EGF in Dermo1+ cells leads to chondroma and cartilage and joint defects. (*A*) Two‐month‐old HB‐EGF overexpression mice showed smaller size (right panel). Western blot results showed that HB‐EGF was highly expressed in BMSCs of *Dermo1‐HB‐EGF* mice (left panel). The lower bands of HB‐EGF may be truncated forms. The quantitation data of the HB‐EGF bands were shown beneath the gel with the value of control being set at 1.0. (*B*) Two‐month‐old *Dermo1‐HB‐EGF* mice had decreased body weight compared with control littermates. *n* = 8. (*C*) Two‐month‐old *Dermo1‐HB‐EGF* mice had decreased body length compared with control littermates. *n* = 8. (*D*) Two‐month‐old *Dermo1‐HB‐EGF* mice had decreased femur length compared with control littermates. *n* = 8. (*E*) Two‐month‐old *Dermo1‐HB‐EGF* mice had increased femur width compared with control littermates. *n* = 8. (*F*) Histology of the knee joint showed severe defects in joint formation in 2‐month‐old *Dermo1‐HB‐EGF* mice. The sections were stained with H/E. Scale bar = 200 µm. (*G*) Safranin O staining showed multiple chondroma‐like lesions around the disordered growth plates of the tibia of 2‐month‐old *Dermo1‐HB‐EGF* mice. Right panel: higher magnification views of areas shown in the boxes. Scale bars = 200 µm (left panel) and 20 µm (right panel). (*H*) Immunostaining of FSP1 at the articular surface of femur bones of 2‐month‐old *Dermo1‐HB‐EGF* and control mice. Scale bar = 50 µm. (*I*) H/E and Ki67 staining of the growth plate of 1‐month‐old *Dermo1‐HB‐EGF* mice and normal mice. Right panels: quantitation data. *n* = 4. Scale bar = 50 µm. (*J*) Immunostaining of Col2 and Col10 at the growth plate and articular cartilage of femur bones in 1‐month‐old *Dermo1‐HB‐EGF* and control mice. Right panels: quantitation data. *n *= 4. Scale bar = 50 µm. (*K*) Histology analysis revealed that 2‐month‐old *Dermo1‐HB‐EGF* mice showed increased defects in growth plate and articular cartilage over time. The femurs of *Dermo1‐HB‐EGF* and control mice at different ages were sectioned and stained with H/E. Scale bars = 200 µm.

To further understand the functions of HB‐EGF, we generated the *Dermo1‐HB‐EGF* overexpression mouse line. To this end, we took advantage of the *ROSA‐STOP‐DTR* mouse line, which has the simian HB‐EGF open reading frame (DTR) inserted into the ROSA26 locus with a floxed stop codon before HB‐EGF.^30^ We crossed *Dermo1‐Cre* to *ROSA‐STOP‐DTR* mice to generate *Dermo1‐HB‐EGF* mice. Cre‐mediated excision of the floxed stop codon will lead to HG‐EGF expression under the promoter of ROSA26 in Dermo1+ cells and their progenies. HB‐EGF is highly conserved and this strategy has been used to express HB‐EGF in mouse intestinal enterocytes and other tissues.^31^ We found that the HB‐EGF levels in bone marrow stromal cultures were elevated in *Dermo1‐HB‐EGF* mice (Fig. 2 *A*). At 2 months of age, the mutant mice were slightly smaller, with a decrease in body weight, body length, and femur length but an increase in femur width (Fig. 2 *A–E*). H/E staining revealed that the mutant mice had distorted knee joints (Fig. 2 *F*).

*Dermo1‐HB‐EGF* mice also developed chondroma near articular cartilage and the growth plate, a benign cartilaginous overgrowth, and fibrosis‐like phenotypes at the articular surfaces, which were stained positive for FSP1, a fibroblast marker, at 2 months of age (Fig. 2 *G*, *H*). All *Dermo1‐HB‐EGF* mice developed chondroma‐like lesions, although to various degrees. Ki67 staining revealed that HB‐EGF overexpression led to an increase in the number of proliferating chondrocytes and an increase in the thickness of growth plate in 1‐month‐old mice (Fig. 2 *I*), suggesting that HB‐EGF promoted expansion of chondrocytes. On the other hand, the mutant mouse bone sections displayed a decrease in the number of cells positive for Col2, a chondrocyte marker, and cells positive for Col10, a hypertrophic chondrocytes marker, at the growth plate and articular cartilage surface (Fig. 2 *J*). These results indicate that HB‐EGF expressed in Dermo1+ cells promoted proliferation but impaired commitment and differentiation of chondrocytes in vivo.

We also examined these mice at different ages and found that the skeleton of the mutant mice was normal at p1 without any change in the size of bone and the morphology of the knee joints (Supplemental Fig. S3*A–C*), suggesting that the cartilage defects observed in adult *Dermo1‐HB‐EGF* mice were developed postnatally. Moreover, we found these cartilage defects, including chondroma, got worse with age (Fig. 2 *K*). At 2 months of age, the skull bones of *Dermo1‐HB‐EGF* mice appeared to be normal (Supplemental Fig. S3*D*). Finally, we found that the vertebrae of *Dermo1‐HB‐EGF* mice also showed similar defects, including chondroma (Supplemental Fig. S4). These results suggest that HB‐EGF affects postnatal skeletal growth but not embryonic development.

### Expression of HB‐EGF in chondrocytes leads to less severe cartilage defects

**Figure 3:**
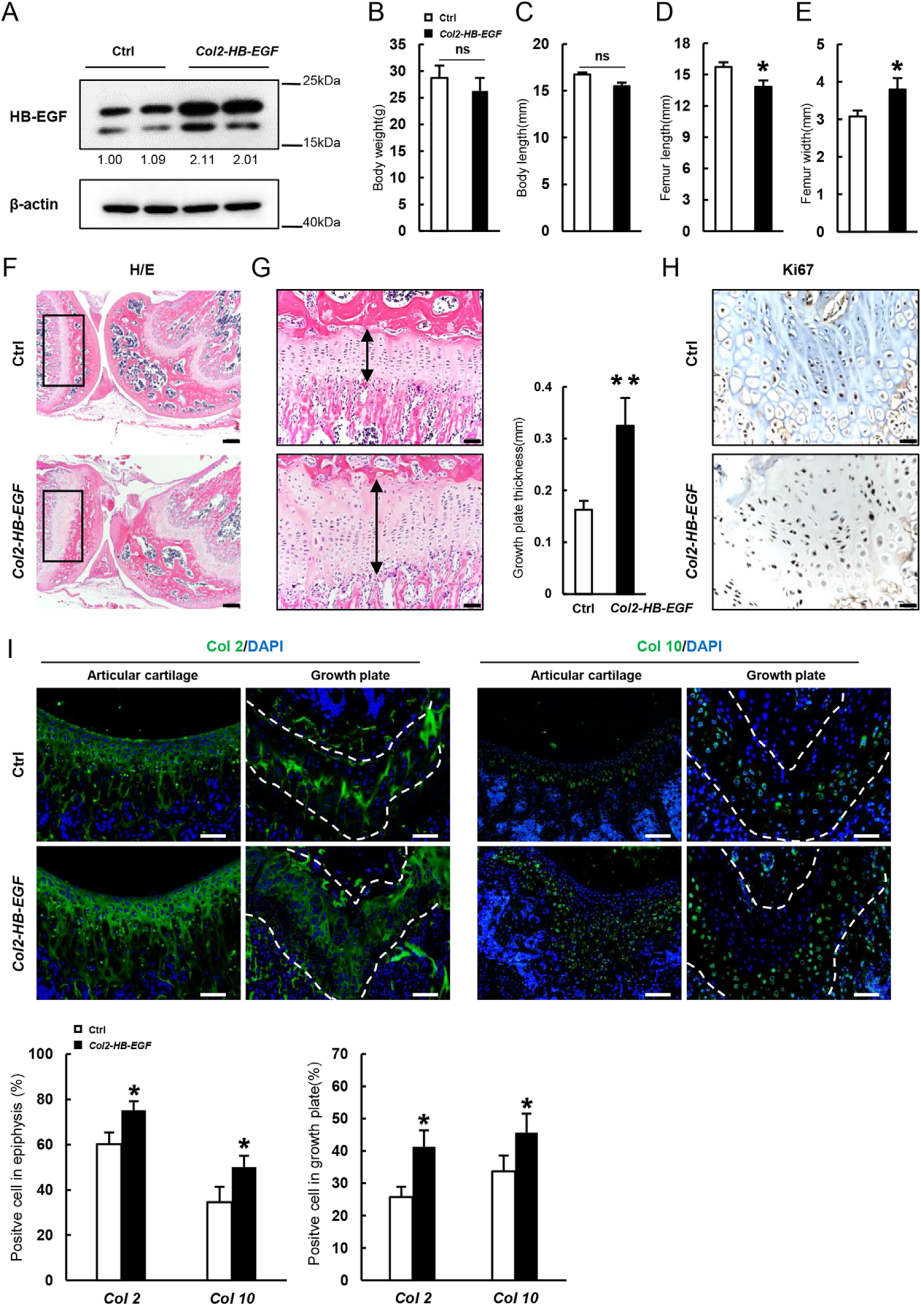
Overexpression of HB‐EGF in chondrocytes leads to modest cartilage defects. (*A*) Western blot results showed that HB‐EGF was highly expressed in cartilage. The epiphysis was dissected out and homogenized for Western blot analysis. The lower bands of HB‐EGF might be truncated forms. The quantitation data of the HB‐EGF bands were shown beneath the gel with the value of control being set at 1.0. (*B*) Two‐month‐old *Col2‐HB‐EGF* mice had normal body weight compared with control littermates. *n* = 4. (*C*) Two‐month‐old *Col2‐HB‐EGF* mice had normal body length compared with control littermates. *n* = 4. (*D*) Two‐month‐old *Col2‐HB‐EGF* mice had decreased femur length compared with control littermates. *n* = 4. (*E*) Two‐month‐old *Col2‐HB‐EGF* mice had increased femur width compared width control littermates. *n* = 4. (*F*) H/E staining revealed that 2‐month‐old *Col2‐HB‐EGF* mice showed a modest defect in the knee joints. The bone sections were stained with H/E. Scale bar = 200 µm. (*G*) Histology analysis revealed that 2‐month‐old *Col2‐HB‐EGF* mice showed an increase in the thickness of the growth plate. Right panel: quantitation data. *n* = 4. Scale bar = 50 µm. (*H*) Immunostaining revealed that 1‐month‐old *Col2‐HB‐EGF* mice showed an increase in Ki67+ chondrocytes in the growth plate. Scale bar = 20 µm. (*I*) Immunostaining revealed that 2‐month‐old *Col2‐HB‐EGF* mice showed a modest increase in Col2‐ and Col10‐positive chondrocytes at growth plates and articular cartilage. Bottom panels: quantitation data. *n* = 4. Scale bar = 50 µm.

The above studies suggest that HB‐EGF promotes chondrocyte proliferation and inhibits their differentiation. To validate this finding, we expressed HB‐EGF in Col2+ chondrocytes by generating *Col2‐HB‐EGF* mice.^32^ HB‐EGF levels were much higher in the femur head of *Col2‐HB‐EGF* mice compared with control mice (Fig. 3 *A*). Two‐month‐old *Col2‐HB‐EGF* mice showed normal body weight and body size but a slight decrease in femur length and an increase in femur width (Fig. 3 *B–E*), as well as distorted knee joints, which were not as severe as those of *Dermo1‐HB‐EGF* mice (Fig. 3 *F*). *Col2‐HB‐EGF* mice did not develop chondroma, yet the growth plate was thicker than control mice, which was associated with an increase in Ki67‐positive chondrocytes (Fig. 3 *G*, *H*). In contrast to *Dermo1‐HB‐EGF* mice, *Col2‐HB‐EGF* mice show a modest increase in the number of cells positive for Col2 and cells positive for Col10 at the growth plate and articular cartilage surface at 2 months of age (Fig. 3 *I*), likely due to increased proliferation of chondrocytes. These results suggest that HB‐EGF has a greater inhibitory effect on chondrogenic commitment than on chondrocyte maturation.

### *Dermo1‐HB‐EGF* mice show a decrease in bone mass and bone formation rate

**Figure 4:**
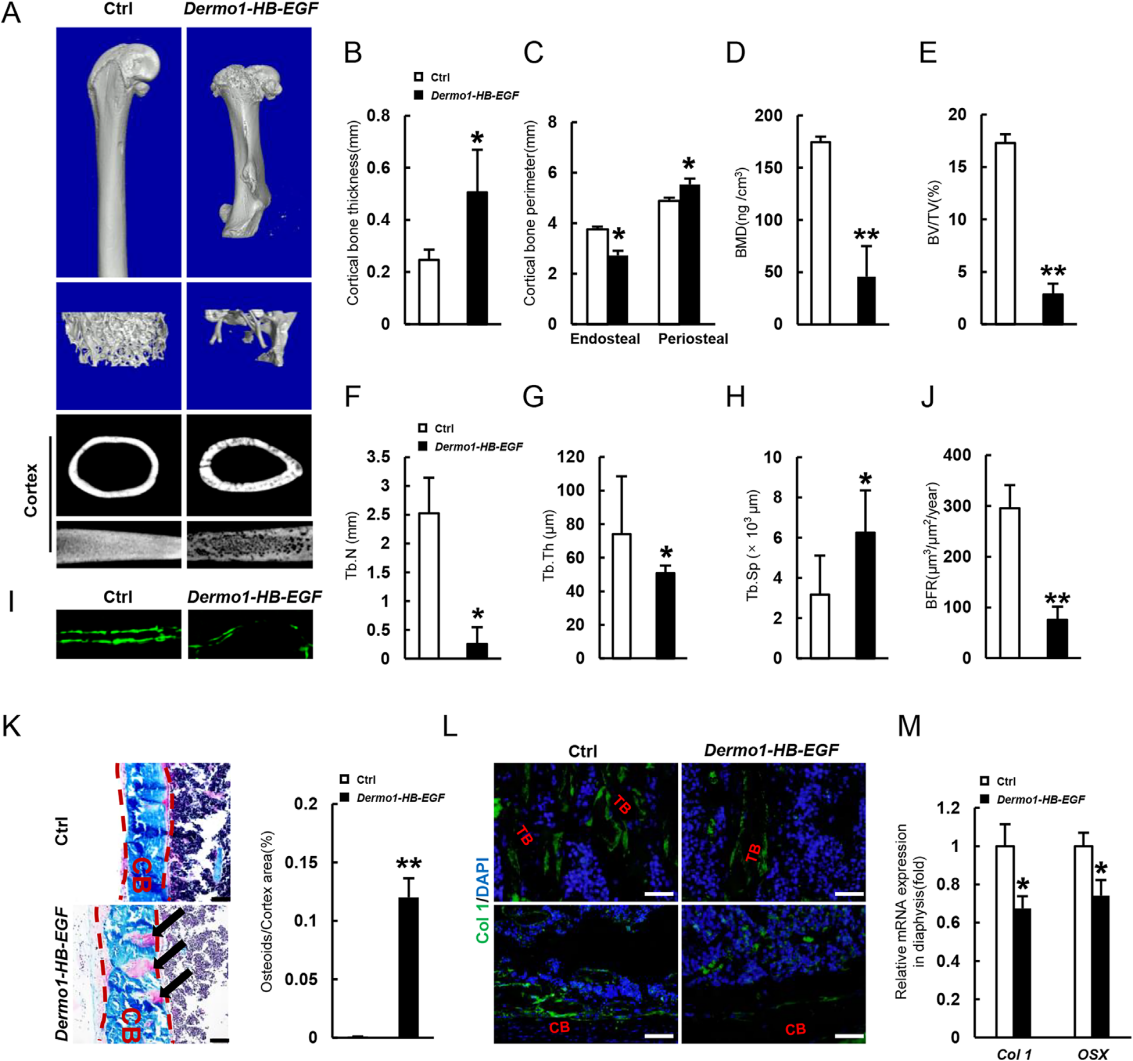
Overexpression of HB‐EGF in Dermo1+ cells leads to a decrease in bone mass associated with osteoblast differentiation and mineralization defects. (*A*) Micro‐CT images of the femurs of 2‐month‐old *Dermo1‐HB‐EGF* and control mice. (*B*) Two‐month‐old *Dermo1‐HB‐EGF* mice showed increased thickness of cortical bones at midshaft. *n* = 8. (*C*) Micro‐CT results of femur endosteal and periosteal circumferences of 2‐month‐old *Dermo1‐HB‐EGF* and control mice at the midshaft. *n* = 8. (*D*) Two‐month‐old *Dermo1‐HB‐EGF* mice showed decreased BMD. *n* = 8. (*E*) Two‐month‐old *Dermo1‐HB‐EGF* mice showed decreased bone volume. *n* = 8. (*F*) Two‐month‐old *Dermo1‐HB‐EGF* mice showed decreased trabecular bone numbers. *n* = 8. (*G*) Two‐month‐old *Dermo1‐HB‐EGF* mice showed decreased trabecular bone thickness. *n* = 8. (*H*) Two‐month‐old *Dermo1‐HB‐EGF* mice showed increased trabecular bone separation. n = 8. (*I*) Two‐month‐old *Dermo1‐HB‐EGF* mice showed a decrease in double labeling of calcein. (*J*) Two‐month‐old *Dermo1‐HB‐EGF* mice showed decreased bone formation rate. *n* = 8. (*K*) Villanueva‐Goldner's trichrome staining of femur sections showed defective mineralization in cortical bone of 2‐month‐old *Dermo1‐HB‐EGF* mice compared with control. Right panel: quantitation data. Scale bar = 20 µm. Arrows indicate osteoids. (*L*) Immunostaining revealed that 2‐month‐old *Dermo1‐HB‐EGF* mice showed a decrease in Col1α+ osteoblasts at the endosteal surface and the trabecular bones. TB = trabecular bone; CB = cortical bone. Scale bar = 20 µm. (*M*) Quantitative PCR results revealed that 2‐month‐old *Dermo1‐HB‐EGF* mice showed decreased expression of Col1α and Osx in the diaphysis. *n* = 4.

We then looked at the bone parameters in 2‐month‐old *Dermo1‐HB‐EGF* mice. The long bones of the mice were shorter and thicker (Figs. 4 *A* and 2*D*, *E*). The thickness of cortical bones was greater than in control mice, which was increased over time (Figs. 4 *B* and 2*K*). The increase in cortical thickness was associated with an increase in periosteal circumference and a decrease in endosteal circumference (Fig. 4 *C*). Micro‐CT results indicated that 2‐month‐old *Dermo1‐HB‐EGF* mice showed a decrease in bone mineral density, bone mass, and the number and thickness of trabeculae but an increase in separation of trabeculae (Fig. 4 *D–H*). Calcein labeling experiments revealed a decrease in bone formation rate in *Dermo1‐HB‐EGF* mice (Fig. 4 *I*, *J*).

Moreover, the cortical bones appeared to be porous (Fig. 4 *A*), indicating a defect in mineralization. Indeed, we found that cortical bones of 2‐month‐old *Dermo1‐HB‐EGF* mice showed 12% osteoid that was not fully mineralized, whereas control mice showed almost no osteoid (Fig. 4 *A*, *K*). Immunostaining and quantitative PCR revealed that femur sections showed a decrease in Col1α and Osterix expression (Fig. 4 *L*, *M*). A decrease in the production of collagen I may contribute to undermineralization of the cortical bones in *Dermo1‐HB‐EGF* mice. These results indicate that HB‐EGF suppressed osteogenic differentiation and mineralization in vivo, which may contribute to the osteoporotic phenotypes observed in *Dermo1‐HB‐EGF* mice.

### *Dermo1‐HB‐EGF* mice show normal osteoclastogenesis and bone resorption

Previous studies have implicated EGFR signaling in coupling bone formation and resorption.^16^, ^33^ However, we found that 2‐month‐old *Dermo1‐HB‐EGF* mice showed no change in the number of TRAP‐positive osteoclasts or bone resorption rate, reflected by normal levels of urine DPD, an in vivo bone resorption marker (Supplemental Fig. S5*A–C*). Furthermore, BMSCs isolated from normal and *Dermo1‐HB‐EGF* mice expressed similar levels of RANKL, OPG, and M‐CSF (Supplemental Fig. S5*D*). We also found that even 50 ng/mL of exogenous HB‐EGF did not show obvious effect on osteoclast differentiation in vitro (Supplemental Fig. S5*E*, *F*). These results, taken together, suggest that HB‐EGF expressed in mesenchymal stromal cells and their progenies does not affect osteoclastogenesis or bone resorption directly or via BMSCs/osteoblasts.

### Inhibition of EGFR with AG1478 rescues the skeletal defects caused by HB‐EGF

**Figure 5:**
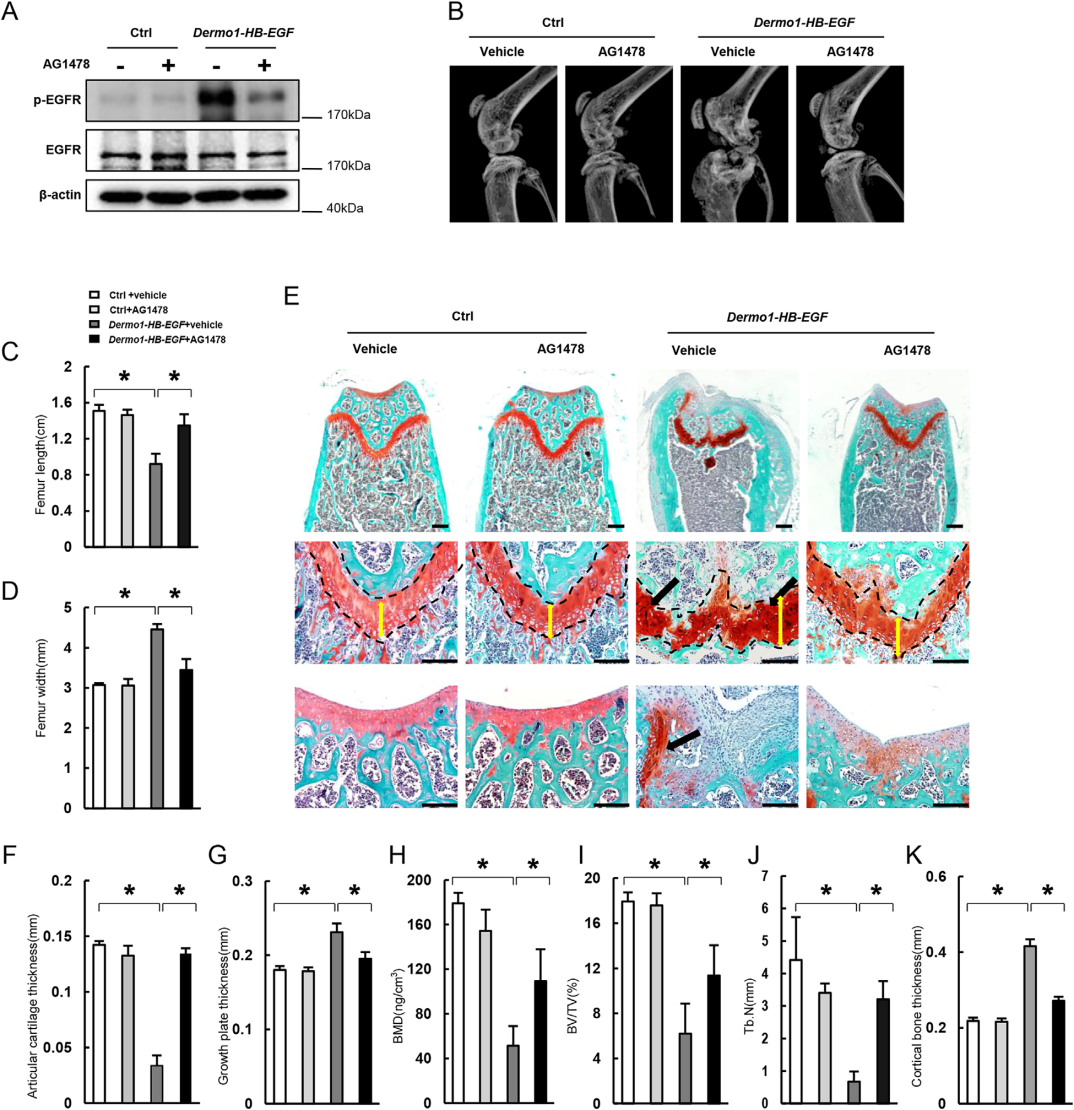
Inhibition of EGFR with AG1478 rescues the cartilage and bone defects of *Dermo1‐HB‐EGF* mice. (*A*) Western blot results showed that AG1478 inhibited EGFR activation in BMSCs of *Dermo1‐HB‐EGF* mice. BMSCs were isolated from mouse bone marrow and were used for Western blot analysis. The mice received AG1478 for 2 months, starting at 1 month of age (for *A–K*). (*B*) AG1478 rescued the abnormal morphology of the knee joint of *Dermo1‐HB‐EGF* mice. (*C*) AG1478 rescued the decrease in femur length of *Dermo1‐HB‐EGF* mice. *n* = 4. (*D*) AG1478 rescued the increase in femur width of *Dermo1‐HB‐EGF* mice. *n* = 4. (*E*) Safranin O staining showed that AG1478 rescued the abnormal growth plate and articular cartilage and chondroma in *Dermo1‐HB‐EGF* mice. Scale bar = 200 µm. (*F*) AG1478 rescued the decrease in the thickness of articular cartilage of *Dermo1‐HB‐EGF* mice. *n* = 4. (*G*) AG1478 rescued the increase in the thickness of growth plate of *Dermo1‐HB‐EGF* mice. *n* = 4. (*H*) AG1478 rescued the decrease in BMD of *Dermo1‐HB‐EGF* mice. *n* = 8. (*I*) AG1478 rescued the decrease in bone volume of *Dermo1‐HB‐EGF* mice. *n* = 8. (*J*) AG1478 rescued the decrease in trabecular bone numbers of *Dermo1‐HB‐EGF* mice. *n* = 8. (*K*) AG1478 rescued the increase in cortical bone thickness of *Dermo1‐HB‐EGF* mice. *n* = 4.

HB‐EGF can bind to EGFR and Erb4 to activate the downstream Erk and Akt pathways. To demonstrate that the cartilage defects of *Dermo1‐HB‐EGF* mice were induced through EGFR/Erb4 signaling, we administrated AG1478, a small molecular compound that mainly targets EGFR, daily for 2 months starting at 1 month of age. Skeletal phenotypes after AG1478 treatment were assessed by X‐ray, micro‐CT, and histological examination. We found that phosphorylation of EGFR was increased in BMSCs of *Dermo1‐HB‐EGF* mice compared with control mice, which were inhibited by AG1478 treatment (Fig. 5 *A*). X‐ray analysis revealed that joint deformation and expansion in *Dermo1‐HB‐EGF* mice were alleviated by AG1478 treatment (Fig. 5 *B*). Moreover, AG1478 largely rescued the cartilage defects caused by HB‐EGF overexpression including femur length and width (Fig. 5 *C*, *D*).

Histological analyses revealed that thinning of Safranin O‐stained articular cartilage in 3‐month‐old *Dermo1‐HB‐EGF* mice was rescued by AG1478 (Fig. 5 *E*, *F*), and so was the increase in growth plate thickness (Fig. 5 *E*, *G*). Furthermore, AG1478 treatment reduced chondromas, especially in trabecular bones (Fig. 5 *E*). These results suggest that HB‐EGF causes cartilage phenotypes via EGFR.

In addition, AG1478 rescued the osteoporotic phenotypes, including the decrease in bone mineral density, bone mass, and cortical bone thickness at 3 months of age (Fig. 5 *H–K*). Taken together, these results indicate that overexpression of HB‐EGF in Dermo1 lineage cells causes skeletal defects and tumorigenesis in an EGFR‐dependent manner.

### HB‐EGF promotes BMSC proliferation but inhibits its differentiation in vitro

**Figure 6:**
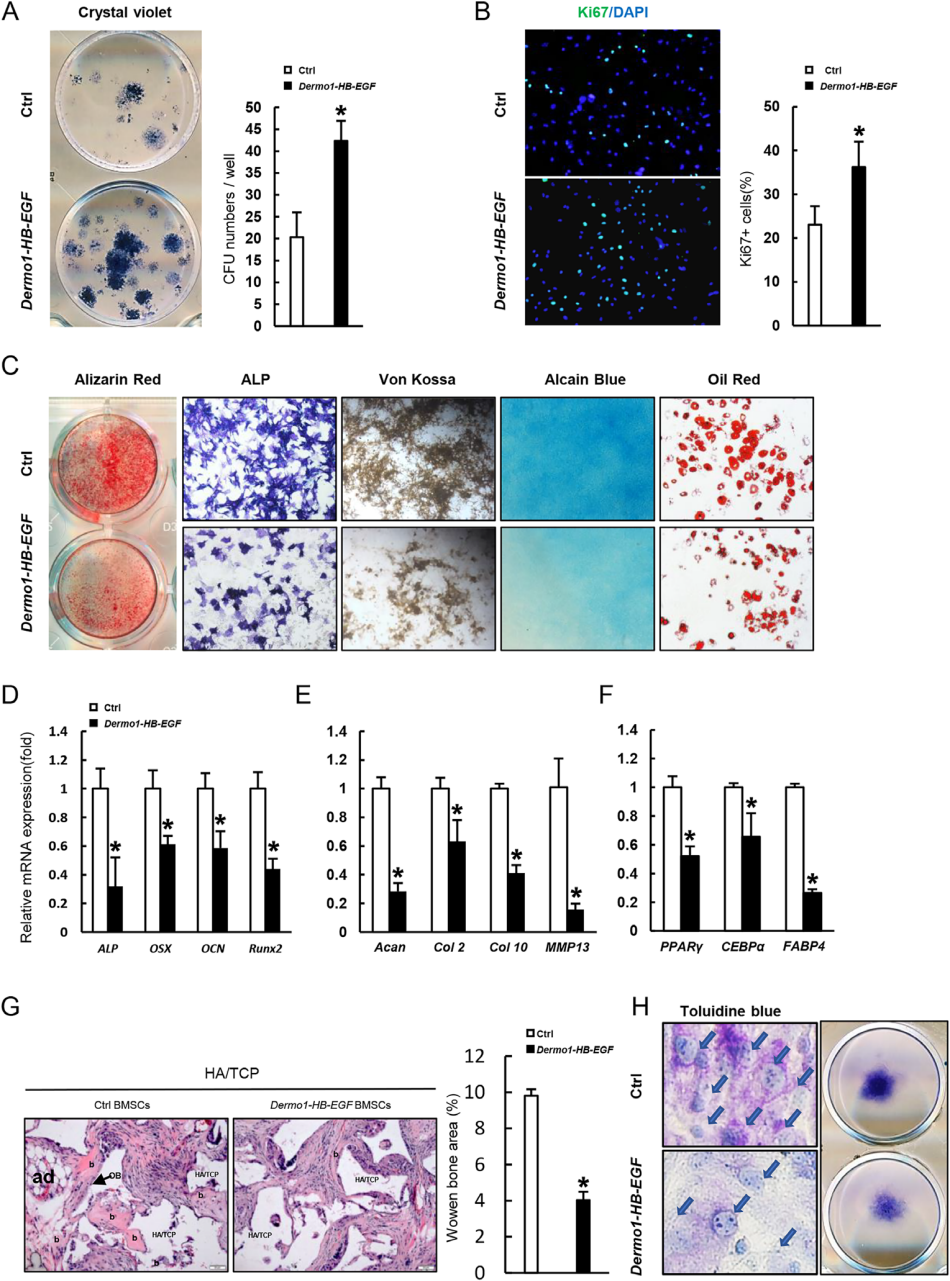
HB‐EGF promotes BMSC proliferation but inhibits differentiation. (*A*) Colony‐forming efficiency assay revealed that 2‐month‐old *Dermo1‐HB‐EGF* mice showed increased numbers of BMSCs that could form colonies. The plates were stained with crystal violet. Right panels: quantitation data. *n* = 3. (*B*) BMSCs isolated from 2‐month‐old *Dermo1‐HB‐EGF* mice showed an increase in proliferation, manifested by an increase in Ki67+ cells. Cells grown at log phase were fixed and stained with anti‐ki67 antibodies. The numbers of Ki67+ cells, which were normalized to the number of nuclei, were presented in the right panel. *n* = 3. (*C*) *Dermo1‐HB‐EGF* BMSCs showed a decrease in osteoblast, chondrocyte, and adipocyte differentiation. Osteoblast differentiation was judged by ALP, Alizarin red, and Von Kossa staining, chondrocyte differentiation was judged by Alcian blue staining, and adipocyte differentiation was judged by Oil Red staining. (*D*) Quantitative PCR revealed that *Dermo1‐HB‐EGF* BMSCs showed decreased expression of osteoblast‐specific markers. The BMSCs were induced to differentiate and then collected at day 5 for qPCR analysis. *n*= 3. (*E*) Quantitative PCR revealed that *Dermo1‐HB‐EGF* BMSCs showed decreased expression of chondrocyte‐specific markers. *n *= 3. (*F*) Quantitative PCR revealed that *Dermo1‐HB‐EGF* BMSCs showed decreased expression of adipocyte‐specific markers. *n* = 3. (*G*) BMSC transplantation experiments revealed that HB‐EGF inhibited BMSC osteogenic differentiation in vivo. HB‐EGF‐expressing Dermo1 lineage BMSCs and control BMSCs with HA/TCP scaffolds were transplanted into nude mice, which were sectioned and stained with H/E 6 weeks later. Arrows indicate sites of bone formation. Right panel: quantitation of the results. b = bone; OB = osteoblasts; ad = adipocytes. *n* = 3. (*H*) Micromass assays revealed that HB‐EGF inhibited BMSC chondrogenic differentiation. The same numbers of HB‐EGF‐expressing Dermo1 lineage BMSCs and control BMSCs were induced to differentiate into chondrocytes and the plates were stained with Toluidine blue 3 weeks later. Arrows indicate chondrocytes.

The above studies indicate that HB‐EGF regulates proliferation and differentiation of chondrocytes and osteoblasts in vivo. We then performed colony‐forming efficiency assays to determine whether elevated HB‐EGF expression altered the number of BMSCs that can form colonies. We flushed out the bone marrow cells and plated them onto culture dishes. After 10 days, the colonies were stained with crystal violet. It was found that HB‐EGF overexpression increased the number of colonies (Fig. 6 *A*). The stromal cells sorted out from *Dermo1‐HB‐EGF; tdTomato* mice also showed increased proliferation rates, manifested by an increase in the percentage of Ki67‐positive cells (Fig. 6 *B*). We also isolated BMSCs and expanded them for 1 to 2 passages and plated them for differentiation assays. Histochemical staining showed that HB‐EGF inhibited BMSC differentiation to osteoblast and chondrocyte, as well as adipocyte to a lesser extent (Fig. 6 *C*), which were validated by quantitative PCR analysis of markers specific for osteoblasts, chondrocytes, or adipocytes (Fig. 6 *D–F*).

We also validate the above findings by treating normal BMSC cultures with exogenous HB‐EGF. We found that exogenous HB‐EGF promoted proliferation of normal BMSCs and inhibited their differentiation into osteoblasts, chondrocytes, and adipocytes (Supplemental Fig. S6*A–C*).

We further validated the effects of HB‐EGF on osteogenesis with BMSC transplantation assays using HA/TCP scaffolds. We sorted out HB‐EGF‐expressing Tomato+ BMSCs from adult *Dermo1‐HB‐EGF; tdTomato* mice and performed transplantation assays. We found that Dermo1 lineage BMSCs showed some osteogenic activity, which was suppressed by HB‐EGF (Fig. 6 *G*). We also validated the effects of HB‐EGF on chondrogenesis using pellet culture assays. We found that Dermo1 lineage BMSCs isolated from adult mice formed pellets and generated cells that were positively stained by Toluidine blue, but not mature chondrocyte, whereas HB‐EGF‐expressing BMSCs were not positively stained by Toluidine blue (Supplemental Fig. S7). These results suggest that HB‐EGF might inhibit BMSC chondrogenic differentiation under this setting and that Dermo1 lineage BMSCs have limited osteogenic and chondrogenic potentials. We also used the micromass assay to test the effect of HB‐EGF on chondrogenesis and found that HB‐EGF‐expressing BMSCs showed decreased chondrogenic activity compared with control cultures (Fig. 6 *H*). Taken together, these results confirm that HB‐EGF has inhibitory effects on BMSC osteogenic and chondrogenic differentiation.

### HB‐EGF inhibits BMSC in vitro osteogenic and chondrogenic differentiation via Smad1 signaling

**Figure 7:**
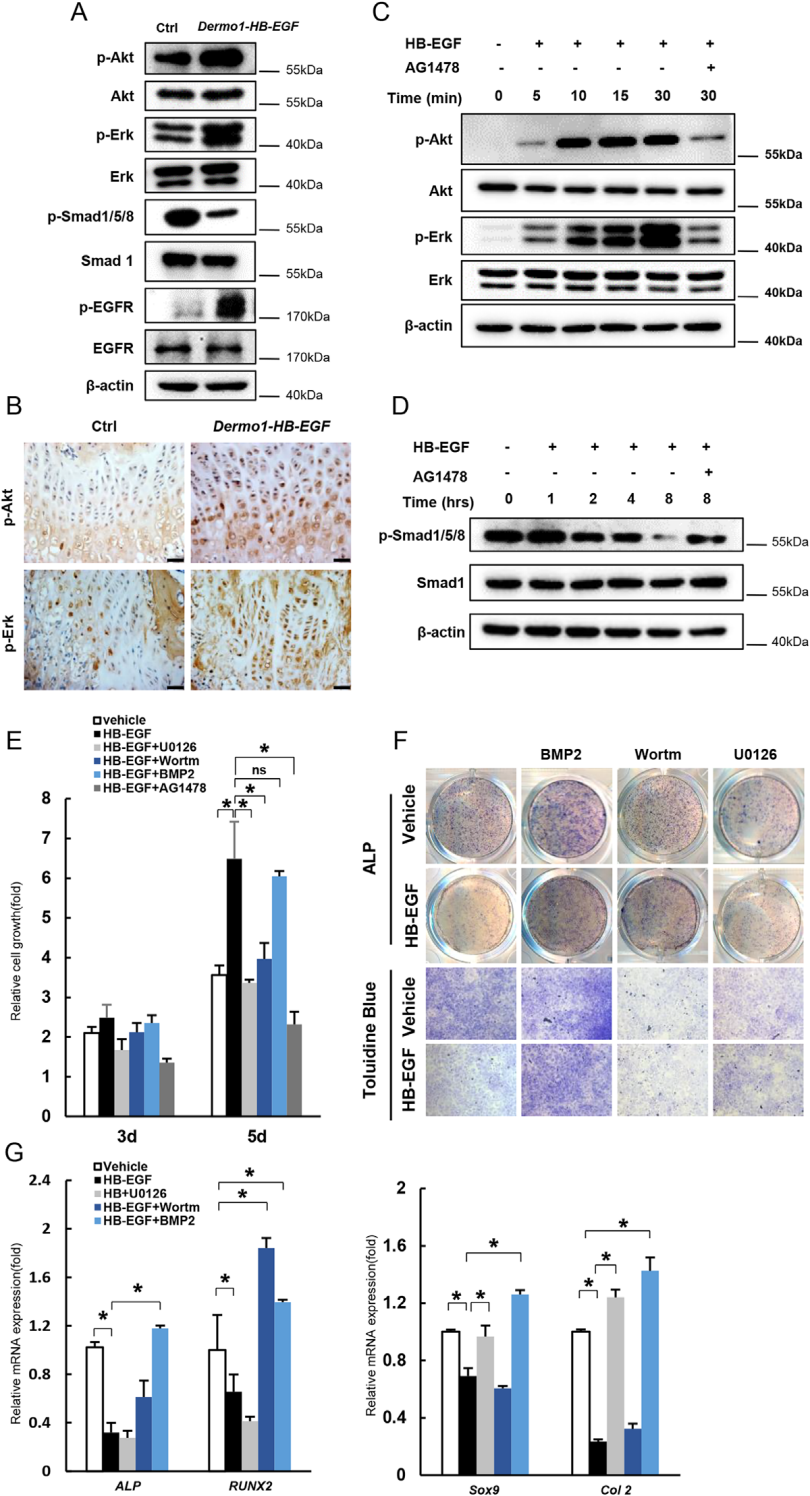
HB‐EGF promotes BMSC proliferation via Akt1/Erk and inhibits differentiation via Smad1 signaling. (*A*) Western blot results indicated that BMSCs isolated from *Dermo1‐HB‐EGF* mice showed enhanced activation of Erks and Akt1 but decreased activation of Smad1/5/8. (*B*) IHC staining confirmed that 2‐month‐old *Dermo1‐HB‐EGF* mouse bone sections showed increased p‐Erk and p‐Akt signals. Scale bar = 20 µm. (*C*) HB‐EGF activated Erk and Akt1 in primary BMSC cultures. BMSCs were treated with 50 ng/mL HB‐EGF and with or without 10 µM AG1478 for 5, 10, 15, or 30 minutes and then harvested for Western blot. (*D*) HB‐EGF inhibited activation of Smad1/5/8 in BMSC cultures. BMSCs were treated with 50 ng/mL HB‐EGF and with or without 10 µM AG1478 for 1, 2, 4, or 8 hours and then harvested for Western blot. (*E*) Inhibition of Erk or Akt1 suppressed HB‐EGF‐driven BMSC proliferation. BMSCs were treated with 50 ng/mL HB‐EGF and with 10 µM U0126, 100 nM Wortmannin, 100 ng/mL BMP2, or 10 µM AG1478. Cell proliferation was analyzed using a CKK8 assay. Data are presented as the mean ± SD. *p < 0.05 and **p < 0.01. *n* = 3. (*F*) BMP2 rescued HB‐EGF‐suppressed BMSC osteogenic and chondrogenic differentiation. BMSCs were cultured in osteogenic or chondrogenic differentiation mediums treated with 50 ng/mL HB‐EGF and with 10 µM U0126, 100 nM Wortmannin, 100 ng/mL BMP2, or 10 µM AG1478. ALP and Toluidine blue staining were performed later. (*G*) Quantitative PCR results revealed that BMP2 rescued HB‐EGF‐suppressed BMSC osteogenic and chondrogenic differentiation. *n* = 3.

To understand the molecular mechanisms by which HB‐EGF regulates BMSC proliferation and differentiation, we compared the activation of HB‐EGF downstream signaling pathways in BMSCs isolated from *Dermo1‐HB‐EGF* and control mice by Western blot. As expected, Erk and Akt1 were activated, following activation of EGFR (Fig. 7 *A*). Immunostaining also confirmed enhanced activation of these two pathways on bone sections of *Dermo1‐HB‐EGF* mice (Fig. 7 *B*). Surprisingly, Smad1/5/8 signaling was suppressed in HB‐EGF‐expressing BMSC cultures (Fig. 7 *A*). The BMP‐Smad1 pathway plays a critical role in osteoblast and chondrocyte differentiation.^34^, ^35^, ^36^, ^37^ Treating BMSC cultures with HB‐EGF activated Erk and Akt pathways and inhibited Smad1/5/8 pathways, although with different kinetics (Fig. 7 *C*, *D*).

To determine which pathway(s) is involved in HB‐EGF‐induced BMSC proliferation, we used inhibitors that specifically target Erks and Akt1 to pretreat BMSC cultures before adding HB‐EGF. HB‐EGF‐driven BMSC proliferation was suppressed by either U0126 or Wortmannin (Fig. 7 *E*), suggesting that these two pathways contribute to HB‐EGF‐driven BMSC proliferation in vitro. In addition, we found that BMP2 could rescue HB‐EGF‐induced defects in BMSC in vitro osteoblast or chondrocyte differentiation (Fig. 7 *F*, *G*). Interestingly, HB‐EGF‐induced suppression of BMSC osteoblast differentiation was rescued by Akt1 inhibitor but not by Erk inhibitor (Fig. 7 *F*, *G*), whereas HB‐EGF‐induced suppression of BMSC chondrocyte differentiation was rescued by Erk inhibitor but not by Akt1 inhibitor (Fig. 7 *F*, *G*). The relationships between Akt1/Erk and Smad1/5/8 in skeletal development warrant further investigation.^38^

## Discussion

In this study, we generated Dermo1+ cell‐specific and chondrocyte‐specific HB‐EGF overexpression mouse models and Dermo1+ cell‐specific *HB‐EGF* knockout mouse model to study the function of HB‐EGF in skeletal development and growth (Supplemental Table S2). We found that *Dermo1‐Cre*‐mediated *HB‐EGF* knockout mice showed normal bone size and articular cartilage, yet they display a modest increase in BMD and bone mass. On the other hand, *Dermo1‐HB‐EGF* overexpression mice show a decrease in BMD, bone mineralization, and bone mass, suggesting that HB‐EGF expressed in Dermo1 lineage mesenchymal stromal cells plays an important role in bone growth. The defect in mineralization, likely due to reduced expression of Col1, may be an important factor in causing osteoporotic phenotypes in *Dermo1‐HB‐EGF* mice. Moreover, expression of HB‐EGF in Dermo1+ cells leads to chondrodysplasia, chondroma, and shortened long bones. HB‐EGF increases proliferation of chondrocytes and osteoblasts but inhibits their differentiation. Because HB‐EGF expression is strong in proliferating cells and downregulated in differentiated cells, we conclude that HB‐EGF not only promotes mesenchymal stromal cell proliferation but also keeps them in an undifferentiated status; this helps mesenchymal stromal cells’ expansion and preservation. Downregulation of HB‐EGF expression in differentiated cells allows for proper maturation of osteoblasts and chondrocytes. Enhanced HB‐EGF activity impairs mesenchymal stromal cell differentiation and reduces the production of functional cells in the skeleton, which cannot be compensated by increase in the numbers of mesenchymal stromal cells. Overall, our findings suggest that HB‐EGF executes its function mainly in the postnatal growth period as *Dermo1‐HB‐EGF* pups appear to be normal right after birth.

Although Dermo1‐Cre can label osteoblasts and chondrocytes during skeletal development and growth, we found that Dermo1 lineage BMSCs isolated from adult mice show limited osteogenic and chondrogenic activities in in vivo ectopic bone formation assay and pellet culture chondrogenesis assay, respectively, compared with studies using the whole BMSCs. These results suggest that the Dermo1 lineage BMSCs act as stem/progenitor cells during skeletal growth but not during bone remodeling in adult mice. Future studies based on Dermo1‐CreERT mice will be needed to prove this point.

We further show that HB‐EGF promotes BMSC proliferation in vitro and this is likely through activating Erk and Akt1 pathways. On the other hand, HB‐EGF‐suppressed BMSC differentiation is likely through inhibiting BMP‐Smad1/5/8 signaling. Interestingly, we found that Akt1 and Erk play different roles in HB‐EGF‐induced BMSC osteogenic and chondrogenic differentiation. Although it is plausible that Erks can phosphorylate Smad1 linker region and thus inhibit Smad1/5/8 activation in response to HB‐EGF,^39^, ^40^ the relationship between Akt1 and Smad1 is elusive. Alternatively, HB‐EGF is known to activate mTOR via Akt1 and mTOR activation has been shown to suppress BMSC differentiation.^28^ Certainly, whether mTOR mediates the effect of HB‐EGF‐Akt1 signaling on BMSC osteogenic differentiation awaits future investigation. Although BMSC may not reflect skeletal stem cells in vivo, the BMSC‐based assays may help to understand the mechanisms by which HB‐EGF regulates proliferation and differentiation of osteoblasts and chondrocytes.

HB‐EGF secreted by tumor cells has been shown to suppress the expression of OPG in osteoblasts, which in turn promotes osteoclastogenesis and produces an environment for tumor cell metastasis.^16^ In contrast, another study shows that expression of a dominant negative EGFR mutant increases the expression of RANKL in osteoblasts, which in turn promotes osteoclastogenesis and leads to a decrease in bone mass.^22^ However, our *Dermo1‐Cre*‐mediated *HB‐EGF* knockout mice and *Dermo1‐Cre*‐mediated HB‐EGF overexpression mice showed no significant change in osteoclastogenesis or bone resorption. Moreover, HB‐EGF‐overexpressing osteoblasts showed no change in expression of RANKL, OPG, or M‐CSF, the coupling factors. Because HB‐EGF can function as paracrine and juxtacrine factors, the above results also suggest that HB‐EGF generated by BMSCs and the progenies may not directly regulate osteoclastogenesis. Indeed, our in vitro assays confirm that exogenous HB‐EGF shows little effect on osteoclast differentiation. These results suggest that HB‐EGF produced in mesenchymal cells and their progenies does not play a direct or indirect role in osteoclastogenesis or coupling bone formation and resorption under normal physiological conditions.

Recent studies have shown that HB‐EGF is involved in development of osteoarthritis, a common disease among the elderly. One study has reported that HB‐EGF levels are increased in osteoarthritis patient samples and HB‐EGF appears to inhibit the anabolic activity but stimulates the catabolic activity of articular chondrocytes.^24^ In contrast, another study showed that overexpression of a dominant negative EGFR led to arthritis in mice.^25^ Our current studies showed that HB‐EGF expression in Dermo1+ cells has severe adverse effects on articular cartilage during postnatal growth, especially on maturation and function of chondrocytes. Thus, HB‐EGF, which is upregulated in osteoarthritis patient samples, may be a growth factor that helps to adapt to stress conditions such as osteoarthritis. Integrating all these findings, we propose that too much HB‐EGF signaling may inhibit differentiation and maturation of articular chondrocytes, whereas too little HB‐EGF‐EGFR signaling may reduce the number of articular chondrocytes, both of which can disrupt the integrity of articular cartilage and leads to development of arthritis‐like phenotypes. As such, the levels of HB‐EGF must be tightly controlled when used for articulate cartilage protection or cartilage engineering.^41^

In sum, our data suggest that HB‐EGF expressed in mesenchymal stromal cells plays critical roles in proliferation and differentiation of osteoblasts and chondrocytes during skeletal growth, in autocrine and/or paracrine fashions. Strong HB‐EGF signaling may impair the integrity of bones and cartilages by suppressing differentiation of chondrocytes and osteoblasts and reducing the production of these cells, causing osteoporotic and osteoarthritis‐like phenotypes. Our observations that *Dermo1‐HB‐EGF* mice show much stronger phenotypes than *Col2‐HB‐EGF* mice and that overexpression of HB‐EGF in chondrocytes does not affect chondrocyte differentiation/maturation indicate that HB‐EGF may play a more important role in chondrocyte progenitors than in differentiated chondrocytes.

## Disclosures

All authors state that they have no conflicts of interest.

## Supporting information

Supporting Figure Legends.

Supporting Figures S1.

Supporting Table S1.

Supporting Table S2.
