# Supplementary material for: Roles for HB‐EGF in Mesenchymal Stromal Cell Proliferation and Differentiation During Skeletal Growth
Source: J Bone Miner Res. 2018 Dec 14;34(2):295–309. doi: 10.1002/jbmr.3596 (PMC7816091; doi:10.1002/jbmr.3596)
Supplement: Supplementary file 1 — Supporting Figure Legends. [file JBMR-34-295-s001.doc]

**Supplementary Table 1. Primer sequences used in the study.**

**Supplementary Table 2. Summary of the major phenotypes of the three mouse lines.**

**Supplementary figure legend**

**Figure S1. HB-EGF expression in bone and bone marrow stromal cells.**

A. ALP staining of BMSC cultures, which were induced to differentiate into osteoblasts and harvested at different time points.

B. ALP, Alcian Blue, and Oil Red staining of BMSC cultures, which were induced to differentiate into osteoblasts, chondrocytes, or adipocytes.

C. HB-EGF was stained positive in the bone. Femur bone sections were immuno-stained with anti-HB-EGF antibodies and FITC-conjugated secondary antibodies. Bottom panels showed the negative control using IgG.

D. Expression of HB-EGF was detected in BMSCs isolated from 2-month-old *Dermo1-Cre; Tomato* mice. BMSCs were isolated and allowed to quickly adhere to coated slide, which were then immuno-stained for HB-EGF. Note that some cells were not labeled by Dermo1, which might be macrophages or other cell types.

**Figure S2. Ablation of *HB-EGF* in Dermo1+ BMSCs leads to a modest increase in bone mass.**

A. Western blot results showed that HB-EGF was deleted in BMSCs of *Dermo1-Cre; HB-EGF^f/f^* mice. The lower bands of HB-EGF might be truncated forms. The quantitation data of the HB-EGF bands were shown beneath the gel with the value of wild type being set at 1.0.

B. Two-month-old *Dermo1-Cre; HB-EGF^f/f^* mice showed normal body weight compared to control littermates. n=4.

C. Two-month-old *Dermo1-Cre; HB-EGF^f/f^* mice showed normal body length compared to control littermates. n=4.

D. Two-month-old *Dermo1-Cre; HB-EGF^f/f^* mice showed normal femur length compared to control littermates. n=4.

E. Micro-CT results showed that 2-month-old *Dermo1-Cre; HB-EGF^f/f^* had normal knee joint.

F. X-ray images showed that 2-month-old *Dermo1-Cre; HB-EGF^f/f^* had normal knee joint.

G. Micro-CT results of the femurs of 2-month-old *Dermo1-Cre; HB-EGF^f/f^* and control mice.

H. Two-month-old *Dermo1-Cre; HB-EGF^f/f^* mice showed a modest increase in BMD compared to control littermates. n=4.

I. Two-month-old *Dermo1-Cre; HB-EGF^f/f^* mice showed a modest increase in BV/TV. n=4.

J. Two-month-old *Dermo1-Cre; HB-EGF^f/f^* mice showed a modest increase in the numbers of trabecular numbers. n=4.

K. Two-month-old *Dermo1-Cre; HB-EGF^f/f^* mice showed no significant change in the thickness of trabecular bones. n=4.

**Figure S3. *Dermo1-HB-EGF* mice show normal knee joint at p1 and normal skull bones in adult mice.**

A. Alizarin Red and Alcian Blue staining of p1 pups.

B. H/E staining of the knee joint of *Dermo1-HB-EGF* and control pups at p1.

C. The lengths of the bones of *Dermo1-HB-EGF* p1 pups were normal.

D. Alizarin Red and Alcian Blue staining of the skulls of 2-month-old *Dermo1-HB-EGF* and control mice.

**Figure S4. *Dermo1-HB-EGF* mice show defects in vertebrae.**

H/E staining and Safranin O staining of the vertebrae sections of 2-month-old *Dermo1-HB-EGF* and control mice. Scale bar: 20 μm.

**Figure S5. Overexpression of HB-EGF in Dermo1+ BMSCs does not affect bone resorption or osteoclastogenesis.**

A. Two-month-old *Dermo1-HB-EGF* mice showed normal TRAP staining on femur bones. Scale bar: 100 μm (upper panel) and 20 μm (bottom panel).

B. Two-month-old *Dermo1-HB-EGF* mice showed normal numbers of osteoclasts. n=8.

C. Two-month-old *Dermo1-HB-EGF* mice showed normal levels of urine DPD. n=8.

D. BMSCs from Two-month-old *Dermo1-HB-EGF* mice expressed normal levels of RANKL, OPG, and M-CSF. n=4.

E. Histochemical staining revealed that HB-EGF did not affect osteoclast differentiation in vitro.

F. Quantitative PCR results showed that HB-EGF did not affect osteoclast differentiation in vitro. n=3.

**Figure S6. Exogenous HB-EGF promotes BMSC proliferation but inhibits its differentiation.**

A. Ki67 staining results showed that HB-EGF promoted proliferation of BMSCs.

B. Histochemical staining showed that HB-EGF inhibited BMSC in vitro differentiation into osteoblasts, chondrocytes, or adipocytes.

C. Real-time PCR results indicate that HB-EGF inhibited BMSC in vitro differentiation into osteoblasts, chondrocytes, or adipocytes. n=3.

**Figure S7. Dermo1 lineage BMSCs isolated from adult mice showed limited chondrogenic activity in cell pellet assays, which was inhibited by HB-EGF.**

A. The pellets formed by Dermo1 lineage BMSCs with HB-EGF overexpressed and control cells.

B. Histochemical staining showed that Dermo1 lineage BMSCs had minimal chondrogenic activity in cell pellet assays, which was inhibited by HB-EGF. The pellets were sectioned and then stained with toluidine blue. n=12.
