# Supplementary material for: Roles for HB‐EGF in Mesenchymal Stromal Cell Proliferation and Differentiation During Skeletal Growth
Source: J Bone Miner Res. 2018 Dec 14;34(2):295–309. doi: 10.1002/jbmr.3596 (PMC7816091; doi:10.1002/jbmr.3596)

Supplemental Figure S1

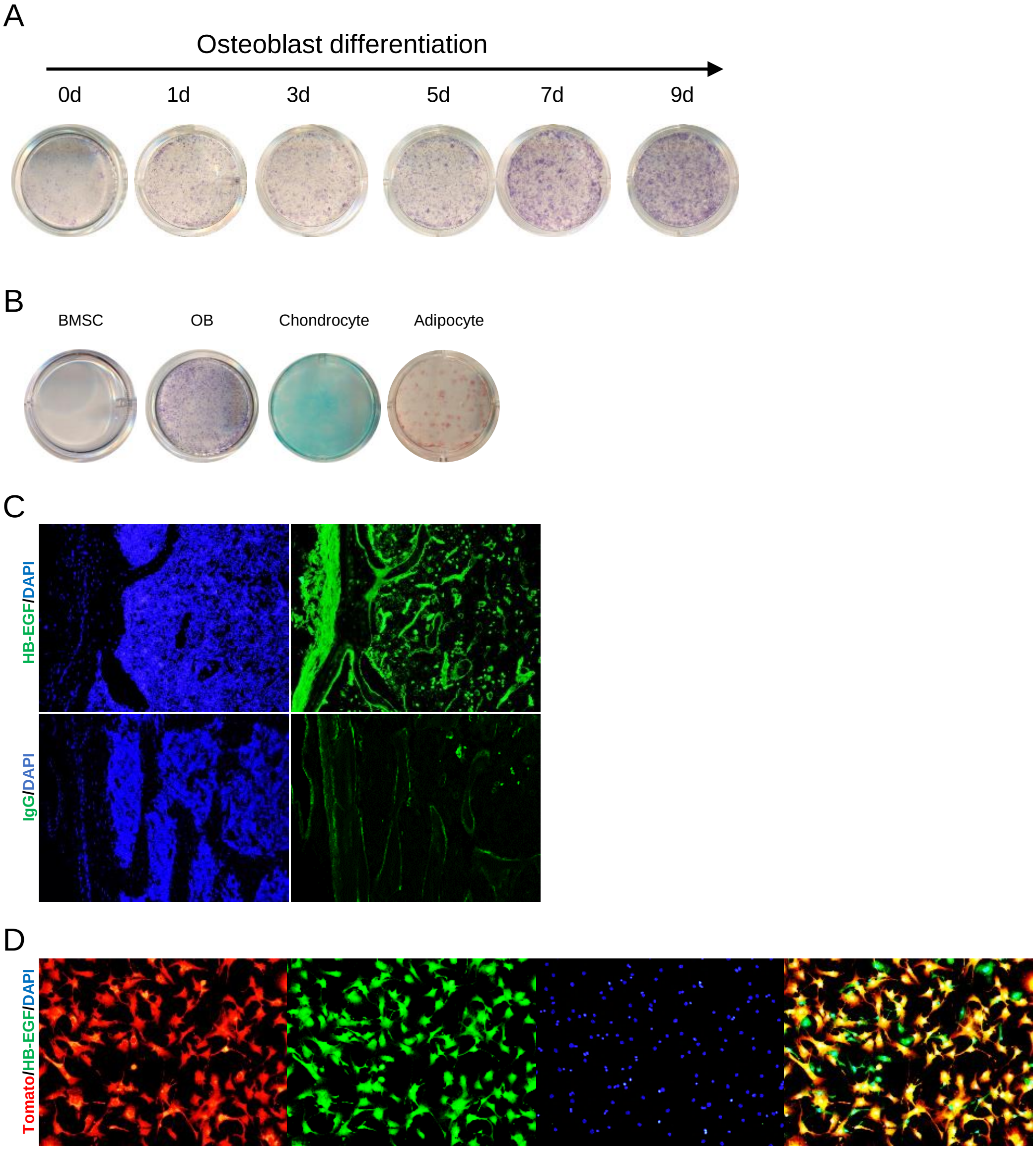

Supplemental Figure S2

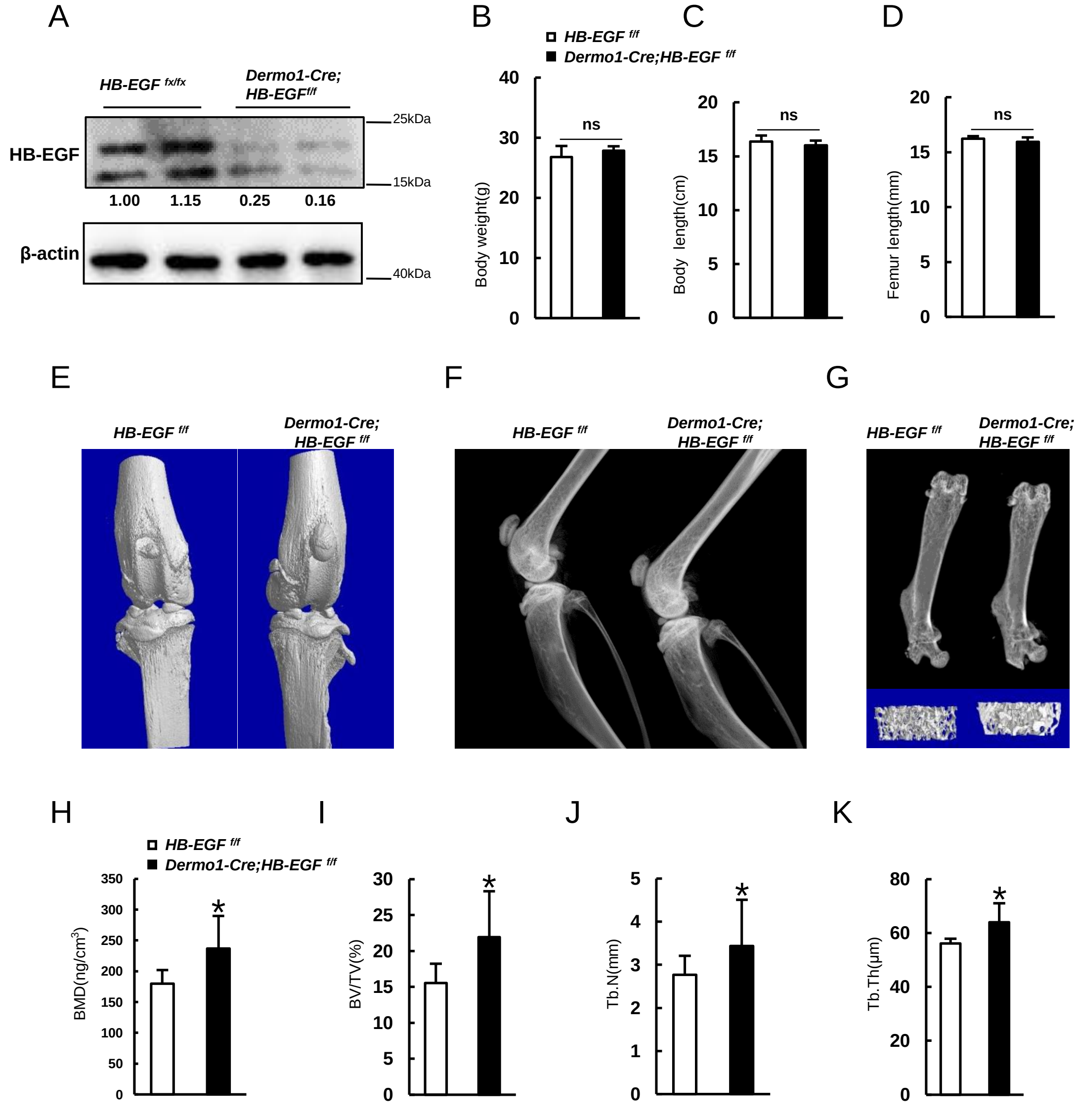

Supplemental Figure S3

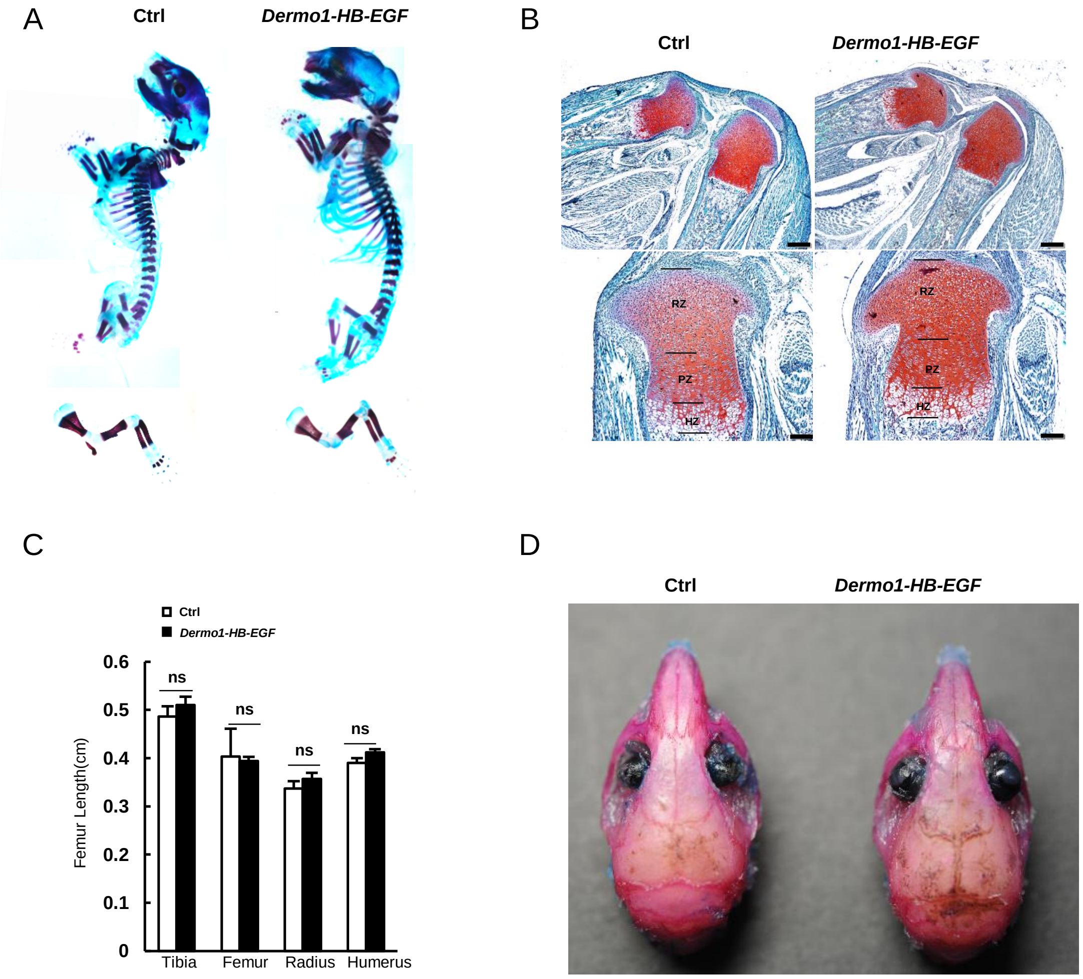

Supplemental Figure S4

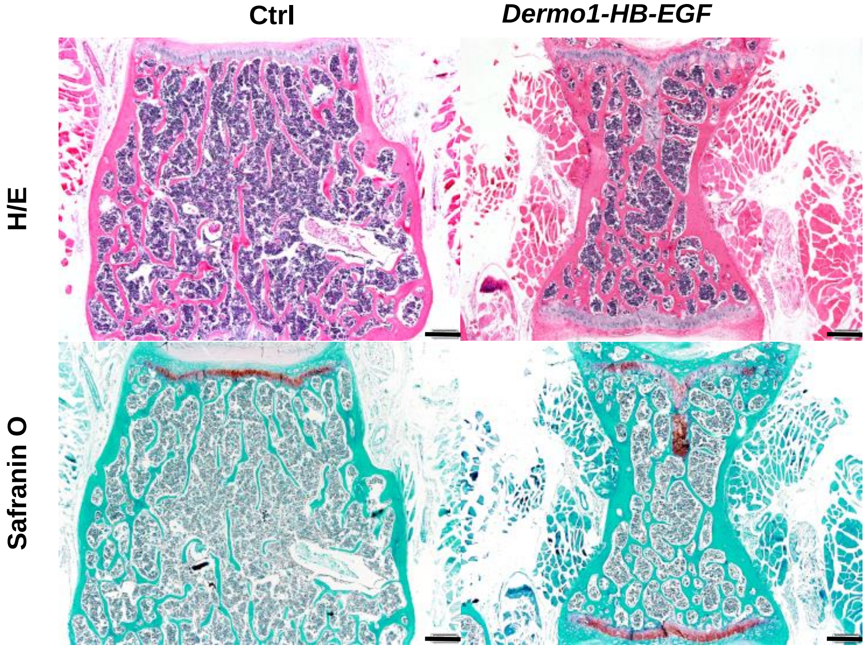

Supplemental Figure S5

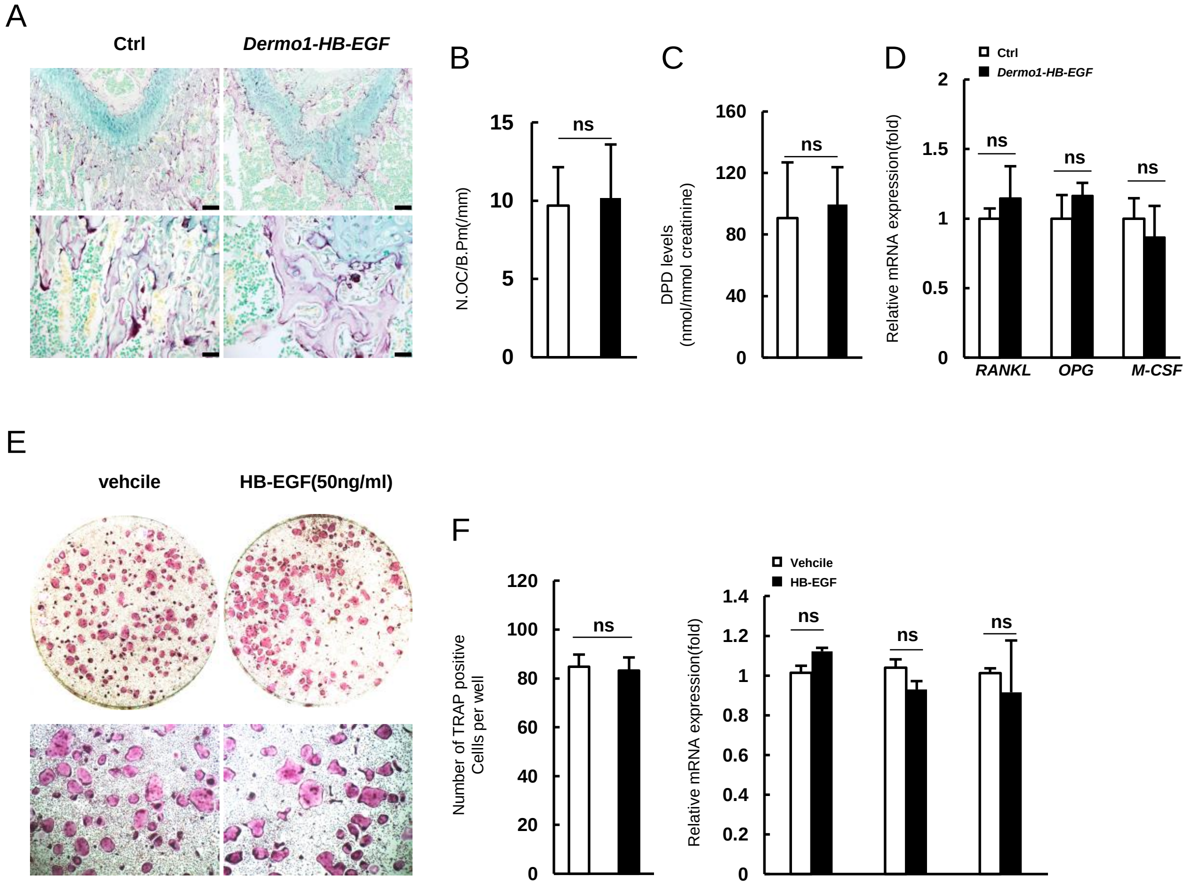

Supplemental Figure S6

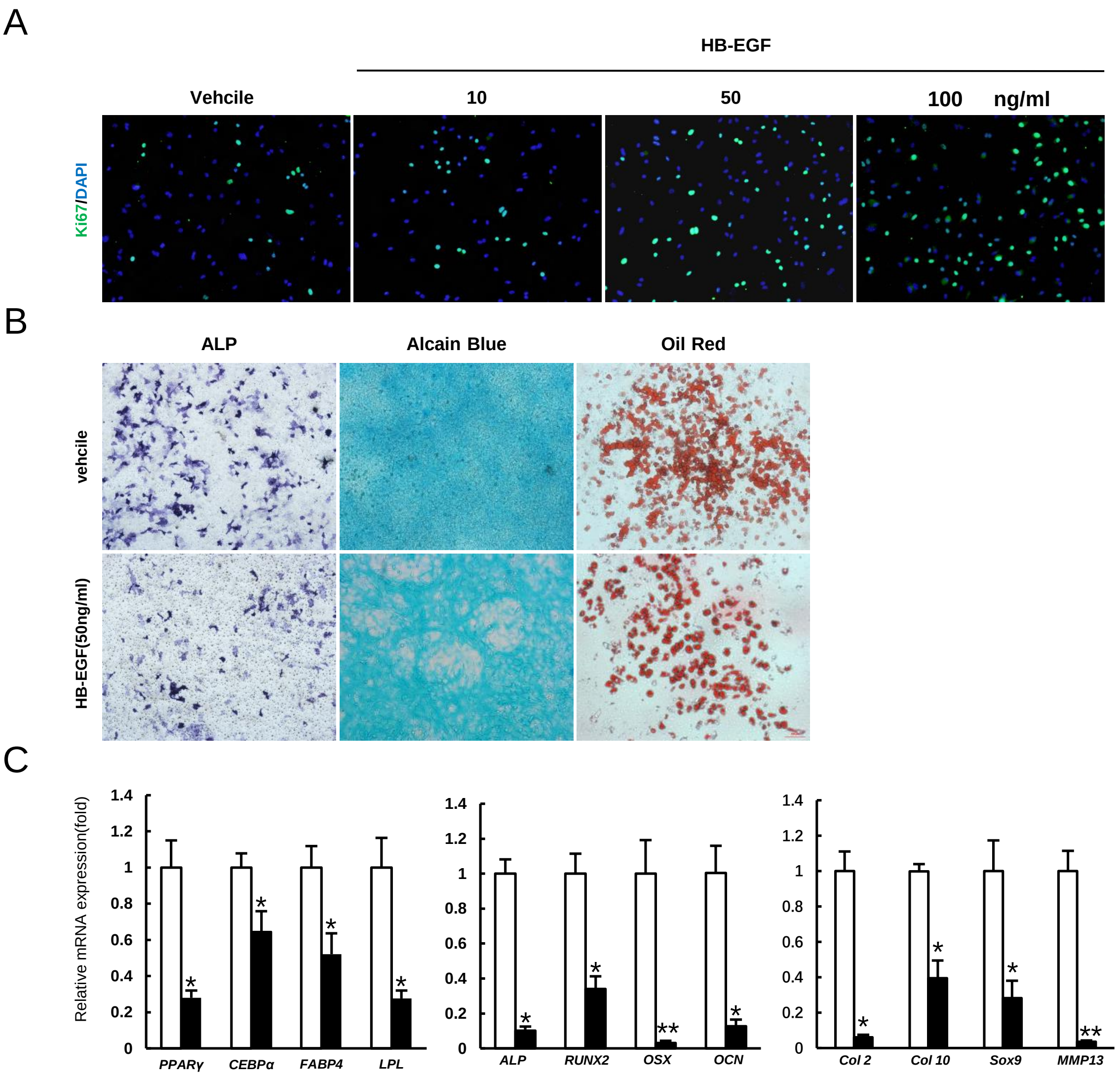

Supplemental Figure S7

A

Ctrl | *Dermo1-HB-EGF*

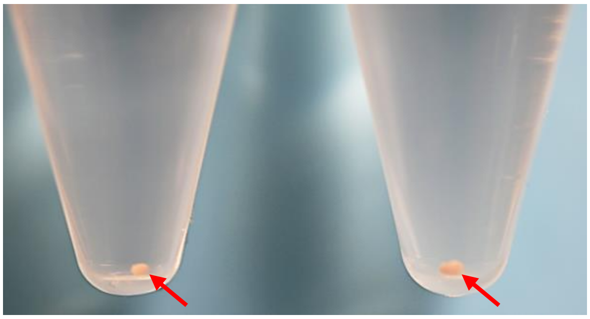

B

Ctrl | *Dermo1-HB-EGF*

Toluidine blue stains

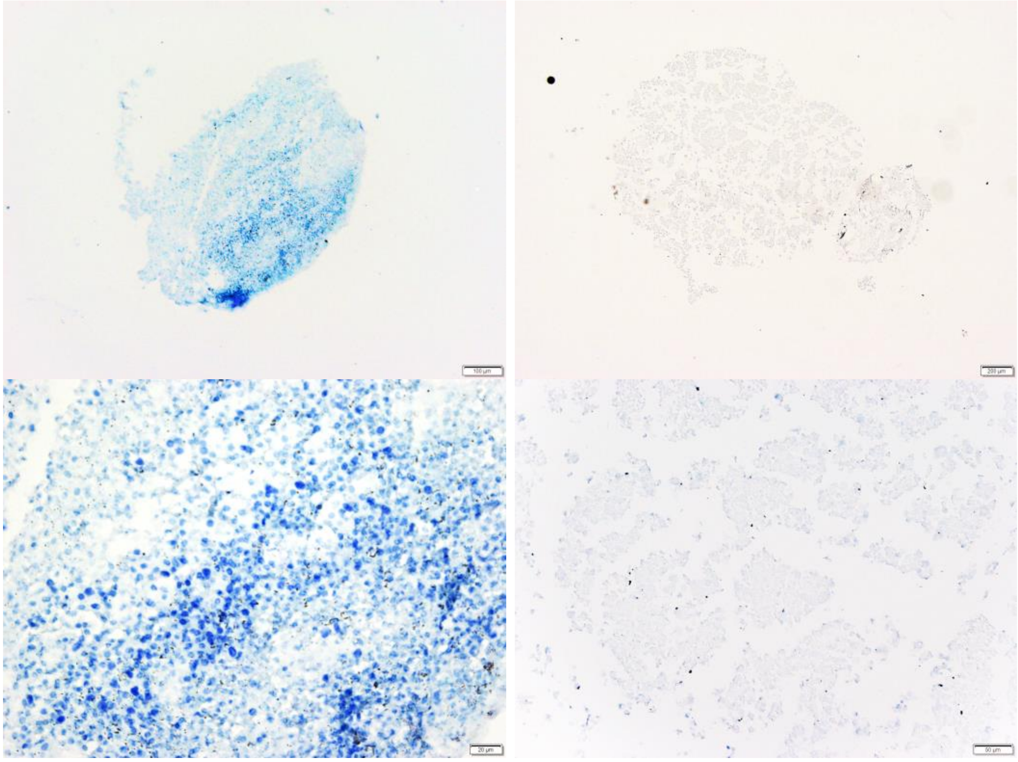

Supplement: Supplementary file 2 — Supporting Figures S1. [file JBMR-34-295-s002.pdf]
