# Supplementary material for: Roles for HB‐EGF in Mesenchymal Stromal Cell Proliferation and Differentiation During Skeletal Growth
Source: J Bone Miner Res. 2018 Dec 14;34(2):295–309. doi: 10.1002/jbmr.3596 (PMC7816091; doi:10.1002/jbmr.3596)
Supplement: Supplementary file 3 — Supporting Table S1. [file JBMR-34-295-s003.doc]

**Supplemental Table S1: Primer sequences used in the study**

| **Gene** | **Forward sequence** | **Reverse sequence** | **Accession Number** |
| --- | --- | --- | --- |
| RUNX2 | GACTGTGGTTACCGTCATGGC | ACTTGGTTTTTCATAACAGCGGA | NM_001146038.2 |
| OSX | ACTCATCCCTATGGCTCGTG | GGTAGGGAGCTGGGTTAAGG | NM_001348205.1 |
| SOX9 | AGTACCCGCATCTGCACAAC | ACGAAGGGTCTCTTCTCGCT | NM_011448.4 |
| MMP13 | TGTTTGCAGAGCACTACTTGAA | CAGTCACCTCTAAGCCAAAGAAA | NM_008607.2 |
| OCN | CAGACAAGTCCCACACAG | GCAGAGTGAGCAGAAAGA | L24431.1 |
| ALP | ATGCCCTGAAACTCCAAA | AGACGCCCATACCATCTC | NM_007431.3 |
| Col2a1 | CCTCAAGGCAAAGTTGGTCCT | CACACGTCTCGGTCATGGTA | NM_031163.3 |
| Col10 | GGGACCCCAAGGACCTAAAG | GCCCAACTAGACCTATCTCA | NM_009925.4 |
| Col1a1 | CGGCTCCTGCTCCTCTTAG | CACACGTCTCGGTCATGGTA | NM_000088.3 |
| CEBPα | GGACAAGAACAGCAACGAG | TCACTGGTCAACTCCAGCAC | NM_007678 |
| FABP4 | GATGAAATCACCGCAGACGACA | ATTGTGGTCGACTTTCCATCCC | NM_024406 |
| PPARγ | GGAAGACCACTCGCATTCCTT | GTAATCAGCAACCATTGGGTCA | NM_001127330.2 |
| LPL | ATGGATGGACGGTAACGGGAA | CCCGATACAACCAGTCTACTACA | NM_008509.2 |
| OPG | TGGGAGAAGAACCTTATTTTG | CCAGCATCCTCTTTCATAAAG | U94331.1 |
| RANKL | GGGAACTGCTGTGGCTTC | GATGGTGAGGTGTGCAAATG | NM_011613.3 |
| M-CSF | GGAGACCTCGTGCCAAATTA | TATCTCTGAAGCGCAAGGTG | BC066205.1 |
| CTSK | GAAGAAGACTCACCAGAAGCAG | TCCAGGTTATGGGCAGAGATT | NM_007802.4 |
| TRAP | TGTCATCTGTGAAAAGGTGGTC | ACTGGAGCAGCGGTGTTATG | NM_001102405.1 |
| NF-ATC1 | TGGAGAAGCAGAGCACAGAC | GCGGAAAGGTGGTATCTCAA | AF239169.1 |
| GAPDH | GGGCTGCTTTTAACTCTGGT | TGGCAGGTTTTTCTAGACGG | NM_002046.5 |
