# Supplementary material for: Roles for HB‐EGF in Mesenchymal Stromal Cell Proliferation and Differentiation During Skeletal Growth
Source: J Bone Miner Res. 2018 Dec 14;34(2):295–309. doi: 10.1002/jbmr.3596 (PMC7816091; doi:10.1002/jbmr.3596)
Supplement: Supplementary file 4 — Supporting Table S2. [file JBMR-34-295-s004.doc]

**Supplemental Table S2: Summary of the major phenotypes of the three mouse lines**

| Mice | phenotypes |
| --- | --- |
| *Dermo1-HB-EGF* | Distorted knee joints; increased femur width; increased growth plate width; chondroma; decreased bone mass |
| *Col2-HB-EGF* | Distorted knee joints; increased femur width; thicker growth plate |
| *Dermo1-Cre;HB-EGF ^f/f^* | Normal skeleton; slight increase in bone mass |
